# Insight into the Mechanism of Action and Peptide‐Membrane Interactions of Aib‐Rich Peptides: Multitechnique Experimental and Theoretical Analysis

**DOI:** 10.1002/cbic.202000834

**Published:** 2021-02-24

**Authors:** Maria Giovanna Lizio, Mario Campana, Matteo De Poli, Damien F. Jefferies, William Cullen, Valery Andrushchenko, Nikola P. Chmel, Petr Bouř, Syma Khalid, Jonathan Clayden, Ewan Blanch, Alison Rodger, Simon J. Webb

**Affiliations:** ^1^ Department of Chemistry University of Warwick Coventry CV4 7AL UK; ^2^ ISIS Neutron and Muon Source Rutherford Appleton Laboratory Harwell Didcot Oxford OX11 0QX UK; ^3^ Department of Chemistry University of Manchester Oxford Road Manchester M13 9PL UK; ^4^ School of Chemistry University of Southampton Highfield Southampton SO17 1BJ UK; ^5^ Manchester Institute of Biotechnology University of Manchester 131 Princess St. Manchester M1 7DN UK; ^6^ Institute of Organic Chemistry and Biochemistry Academy of Sciences Flemingovo náměstí 2 16610 Prague 6 Czech Republic; ^7^ School of Chemistry University of Bristol Cantock's Close Bristol BS8 1TS UK; ^8^ School of Science RMIT University GPO Box 2476 Melbourne Victoria 3001 Australia; ^9^ Department of Molecular Sciences Macquarie University Sydney NSW 2109 Australia

**Keywords:** antimicrobial peptides, lipid-peptide interactions, membranes, molecular dynamics, peptaibols, transfer of chirality

## Abstract

The increase in resistant bacterial strains necessitates the identification of new antimicrobial molecules. Antimicrobial peptides (AMPs) are an attractive option because of evidence that bacteria cannot easily develop resistance to AMPs. The peptaibols, a class of naturally occurring AMPs, have shown particular promise as antimicrobial drugs, but their development has been hindered by their mechanism of action not being clearly understood. To explore how peptaibols might interact with membranes, circular dichroism, vibrational circular dichroism, linear dichroism, Raman spectroscopy, Raman optical activity, neutron reflectivity and molecular dynamics simulations have been used to study a small library of peptaibol mimics, the Aib‐rich peptides. All the peptides studied quickly partitioned and oriented in membranes, and we found evidence of chiral interactions between the phospholipids and membrane‐embedded peptides. The protocols presented in this paper open new ground by showing how chiro‐optical spectroscopies can throw light on the mechanism of action of AMPs.

## Graphical abstract

**Sit up or lie down**: Linear dichroism, circular dichroism and Raman spectroscopy, neutron reflectivity and molecular dynamics simulations have been used to explore how mimics of peptaibol antibiotics (Aib foldamers) interact with membranes. These studies revealed all peptides partitioned well into the bilayers, with orientation dependent upon peptide length, as well as chiral interactions between peptides and the phospholipids.

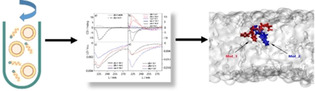

## Introduction

The activity of antimicrobial peptides (AMPs) usually depends upon their ability to bind to a cell membrane and their subsequent interactions with it. The majority of AMPs have a net positive charge and higher percentage of hydrophobic amino acids than globular proteins.^[1]^ However, a few exceptions can be found, such as the peptaibols, soil‐borne AMPs that are extracted from *Tricoderma* fungi. Distinctive features of peptaibols are their high hydrophobicity, zero net charge, modification of both N and C termini, and a high proportion of non proteinogenic amino acids. These amino acid residues, primarily α‐aminoisobutyric acid (Aib) along with isovaline and hydroxyproline, are known to induce the helical folding of the peptides that is thought to be crucial for their antimicrobial activity. Moreover, the nature of the C‐terminal group can greatly affect antimicrobial activity and toxicity against human cells.^[2]^ However, a general mechanism of action for the peptaibol class of AMPs is yet to be understood.

It has been proposed that peptaibols mainly act by modifying membrane permeability, either by forming transmembrane pores/channels or by acting as ion carriers. The archetypical peptaibol alamethicin is know to form voltage‐dependent channels,^[3]^ which are proposed to form through a “barrel‐stave” mechanism involving the association of single peptides into bundles that penetrate the bilayer.^[4]^ Despite numerous studies that aimed to relate peptide length to antimicrobial activity, this relationship is still unclear. The activity of the shorter peptaibols is thought to be facilitated by the assembly of the peptides into supramolecular structures in the membrane.^[5]^ A related class of peptide, comprising short synthetic Aib‐rich foldamers, also display membrane activity and have been employed to mimic membrane‐active molecules, including peptaibols.[^6^, ^7^] The high content of Aib residues in these foldamers leads to the adoption of both *P* (right‐handed) and *M* (left‐handed) 3_10_ helical conformations, and their high hydrophobicity is similar to that of the naturally occurring peptaibols.[^5^, ^8^, ^9^, ^10^]

Understanding the orientation, partitioning and location of peptides located on or in the bilayer of cell membranes is crucial for understanding their antimicrobial activities. However, direct measurement is challenging. Current methods include equilibrium dialysis, differential scanning calorimetry, membrane filtration, chromatography, fluorescence and FRET,^[11]^ all of which usually require modification of either peptide or lipid.[^12^, ^13^, ^14^, ^15^, ^16b^] Solid‐state nuclear magnetic resonance (ss‐NMR) spectroscopy and circular dichroism (CD)^[16a]^ have been used, but as yet provide no clear idea why some peptaibols are active and some are not. We speculated that the membrane insertion geometry was the key, and chose to focus on some relatively short Aib foldamers *without* reported antimicrobial activity to understand why they fail to show activity. Nonetheless, several of these short foldamers have been shown to insert into membranes, and in some cases were reported to show ionophoric activity in vesicle membranes at high concentrations (60–100 μM).[^9^, ^17^, ^18^]

Our focus was on understanding the orientation of Aib rish peptides in lipid membranes using linear dichroism (LD). We also investigated the effect of the peptides on the membrane structure using neutron reflectivity^[5]^ as well as the interplay between peptides and lipid chirality. All of this was complemented by molecular dynamics (MD) simulations to interpret the experimental data. Unlike CD, which can report on peptide helicity, LD (the differential absorption of parallel and perpendicular polarized light by an oriented system) gives information regarding the orientation of the peptides in the membrane.[^19^, ^20^, ^21^, ^22^, ^23^] Like CD, Raman optical activity (ROA) can report on peptide helicity and the first ROA measurement on peptide/liposome mixtures is reported. Despite a common assumption that the enantiomers of membrane‐active peptides have identical activity, studies of the enantiomers peptide kalata B1 interacting with a range of cell lines showed that the bioefficacy of this peptide is modulated by the lipid bilayer chirality.[^1e^, ^1f^] This observation was confirmed by surface plasmon resonance (SPR) and NMR spectroscopic studies of kalata B1 analogues interacting with model phospholipid bilayers, which showed that the insertion into the bilayer is modulated by the chiral environment created by the phospholipids. Similarly, studies on the human defensins HNP1 and HD5 and their respective enantiomers showed that enantiomeric peptides had identical activity against *Escherichia coli*, but the l forms were more potent against *Staphylococcus aureus* than their enantiomers.^[1g]^ These studies suggest that lipid chirality might play a key role in the activity of small membrane targeting peptides.[^1e^, ^1f^, ^1g^]

**Figure 1:**
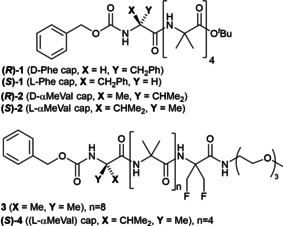
Foldamers **1** and **2** are chiral and bear, respectively, an N‐terminal phenylalanine or an N‐terminal α‐methylvaline. Foldamer **3** is achiral, whereas foldamer (*S*)‐**4** has an N‐terminal (*S*)‐α‐methylvaline.

The focus of this study was on the enantiomeric peptides (*R*)‐**1** and (*S*)‐**1** (Figure 1) which have a hydrophobic surface, an N‐terminal phenylalanine residue capped with a benzyloxycarbonyl (Cbz) group and a C terminus functionalized with a *tert*‐butyl ester that introduces a Schellman‐like motif.^[24]^ The N‐terminal Cbz group functions as an extra hydrogen bond acceptor that stabilizes the 3_10_ helix conformation, and also has the potential to engage in π‐π stacking interactions.[^8^, ^18^, ^25^] We complemented the **1** enantiomers with (*R*)‐**2**, (*S*)‐**2** and (*S*)‐**4**, which each have an N‐terminal α‐methylvaline cap, and **3** which is achiral but long enough to span the hydrophobic region of a membrane.[^25^, ^26^]

Studies in solution have demonstrated that Aib foldamers weakly self‐associate in chloroform solution, a solvent that is often assumed to mimic the membrane environment.[^6^, ^26^] X‐ray investigations on the racemic mixtures of both **1** and **2** showed that in the solid state the peptides adopt a 3_10_ helical conformation.[^18^, ^27^] 8‐Hydroxypyrenetrisulfonate (HPTS) studies of the ionophoric activity of the peptide **1** in EYPC/cholesterol vesicles showed that at concentrations of 60 μM (25 : 2 lipid/peptide), the peptide allowed the leakage of ions and dye through the membrane without disrupting it. This ionophoric activity was found to be higher for the racemate compared to the enantiopure peptides.^[18]^ Based on these results it has been suggested that the peptides exhibit membrane activity through an “amyloid‐like” mechanism with the formation of heterogeneous populations of aggregate that can span the membrane. According to this proposed model, following initial partitioning of the peptides in the membrane they aggregate into a solid‐state‐like assembly with columns of peptides of the same screw‐sense interacting with columns of the opposite screw sense.^[18]^ However, little is known about the propensity of these peptides to aggregate in the membrane, the orientation of the peptides when membrane‐embedded, and the interactions of each peptide enantiomer with the chiral lipids in the bilayer. Herein we show how multiple complementary spectroscopic techniques can be used together to provide insight into all these processes without the need to alter (label) the structure of these small peptides.

## Results and Discussion

### Orientation and partitioning of Cbz(αMeVal)Aib_4_O^t^Bu enantiomers: (*S*)‐2 and (*R*)‐2

**Figure 2:**
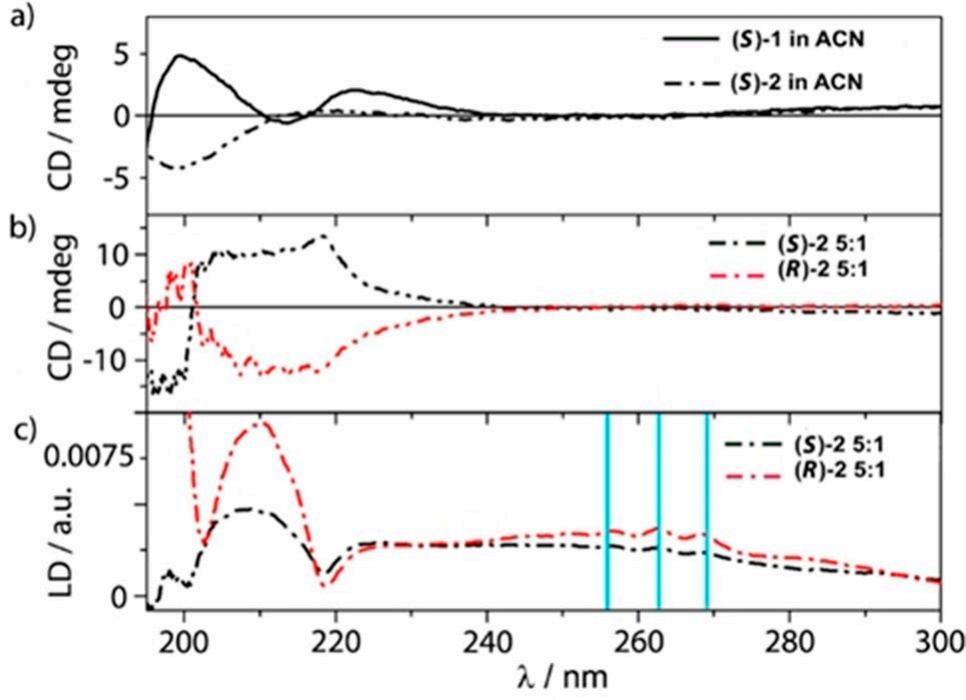
a) CD spectra of (*S*)‐**2** (‐**‐⋅–)** and (*S*)‐**1** (**—**) in acetonitrile (ACN) at 0.1 mg/mL concentration. b) CD and c) LD spectra of enantiomers **2** (**–⋅–**: (*R*)‐**2**, **–⋅–**: (*S*)‐**2**) with liposomes at a lipid/peptide ratio of 5 : 1 (DOPC lipid concentration=5 mg/mL). Positive vibronic signals indicated with blue lines.

*Circular dichroism*: Peptides (*S*)‐**2** and (*R*)‐**2** have a similar hydrophobic surface with the same terminal functionalisation as (*S*)‐**1** and (*R*)‐**1** (which are the peptides of particular interest to this work) but with no N‐terminal aromatic amino acid (only an N‐terminal Cbz group). They are, thus, a good starting point to understand the behaviour of (*S*)‐**1** and (*R*)‐**1** in lipid bilayers. Figure 2a shows the CD spectra of (*S*)‐**2** and (*S*)‐**1** in acetonitrile (ACN). The CD spectrum of (*S*)‐**2** shows a negative band at 200 nm and is similar to the CD of a Aib foldamers in methanol that adopts a right‐handed 3_10_ helical structure,^[28]^ whereas (*S*)‐**1** has a positive signal at 200 nm as well as an excitonic looking signal from 210 nm to 230 nm. Upon addition to 1,2‐dioleoyl‐*sn*‐glycero‐3‐phosphocholine (DOPC) vesicles, (*S*)‐**2** and (*R*)‐**2** have close to enantiomeric CD spectra but with significantly more intensity in the n–π* region (220 nm) than was observed in acetonitrile (Figure 2b). The l‐α‐methylvaline residue at the N terminus of (*S*)‐**2** is known to induce a preference for a right‐handed 3_10_ helix conformation in methanol, with the opposite occurring for d‐α‐methylvaline (as in (*R*)‐**2**).^[29]^ There is a sign change of the CD band at 220 nm from (*S*)‐**2** in acetonitrile compared to (*S*)‐**2** in lipid. However, it is commonly observed for the 220 nm CD band in 3_10_ helical Aib foldamers to change in different solvents or even with concentration.

**Figure 3:**
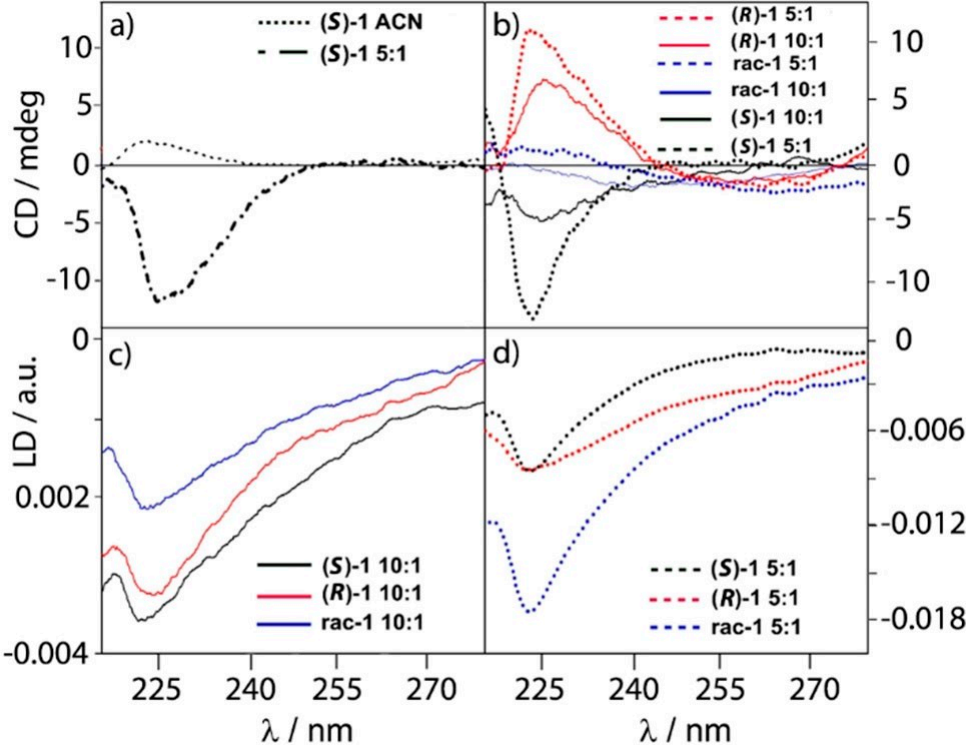
a) CD of (*S*)‐**1** in acetonitrile at 0.1 mg/mL (**⋅⋅⋅⋅**) and in vesicles at a 5 : 1 ratio (5 mg/mL lipid concentration, **‐ ‐ ‐ ‐**). b) CD of (*S*)‐**1** (black), (*R)*‐**1** (red), *rac*‐**1** (blue) at 10 : 1 DOPC lipid/peptide ratio (solid lines) and 5 : 1 (**‐ ‐ ‐ ‐**) lipid/peptide ratio. c), d) LD of (*S*)‐**1** (black), (*R)*‐**1** (red), *rac*‐**1** (blue) at lipid/peptide ratios of 10 : 1 (**—**) and 5 : 1 (**‐ ‐ ‐ ‐**; each 5 mg/mL lipid).

*Linear dichroism*: The LD spectra are very similar in shape but differ by a factor of two in magnitude, suggesting that either (*R*)‐**2** orients better in the membrane or it inserts more effectively than (*S*)‐**2**. Since peptides (*S*)‐**2** and (*R*)‐**2** in bilayers show CD spectra of similar magnitude (if opposite sign, see Figure 2b), the stronger LD signal from (*R*)‐**2** in the bilayer suggest that the orientation of this enantiomer is different, for example more tilted from the vertical normal to the membrane. Such differences between enantiomers must be due to diastereomeric interactions with the chiral membrane environment.[^1e^, ^1f^, ^1g^] The LD of a chromophore inserted in a liposome is given by:

(1) $$\mathrm{LD}=-3/4S(3\;\cos{}^{2}\beta -1)$$

where *S* is the orientation parameter (0 for random and 1 for perfect) and β is the angle between the normal to the membrane and the transition polarisation.[^21a^, ^30^] The negative maximum at ∼220 nm, presumably corresponding to the helix net n‐π* transition that is perpendicular to the helix axis, is consistent with the peptide orienting with respect to the membrane in a manner that is more parallel than perpendicular to the surface. If we assume that all of the peptides are membrane bound, the liposomes have an orientation factor of 0.03, and the peptides absorbance may be taken from Equation (1), then we conclude that (*S*)‐**2** (Figure 2) and (*S*)‐**1** (Figure 3) both have the peptide backbone tilted away from the membrane surface by up to 40°.[^21a^, ^21b^] Any peptide yet to insert into the bilayer contributes to the absorbance but not the LD, so exaggerates our estimate of the tilt. The molecular dynamics simulations (outlined below and summarised in Figure 8) indicate less than a 40° tilt of the backbone and are consistent with these LD data. The positive vibronic signals between 250 nm and 270 nm (Figure 2, blue lines) as well as the positive signals at 210 nm, which we assume belong to the Cbz phenyl group short and long axes respectively, indicate that the plane of the phenyl group of both enantiomers is more parallel to the surface than to the lipid tails.

### Orientation and partitioning of Cbz(Phe)Aib_4_O^t^Bu enantiomers: (*S*)‐1 and (*R*)‐1

*Circular dichroism*: The CD of the peptides **1** in acetonitrile (only (*S*)‐**1** shown, Figure 3a) show more structure in the 220 nm region than the peptides **2**. However, as was the case for peptides **2**, a sign change is observed when the (*S*)‐**1** peptide is in a lipidic environment (Figure 3a), the signs of the 220 nm region for (*S*)‐**1** in a bilayer are the opposite from (*S*)‐**2** in a bilayer and (*S*)‐**1** in ACN. At the same concentration the two enantiomers have CD signals of opposite sign but similar magnitude. Indeed, the racemic mixture only gives a very weak CD signal, which suggests diastereo‐induction by the chiral bilayer is relatively weak in this case; the CD of *rac*‐**1** at the two different peptide ratios (Figure 3b) shows only scattering and perhaps some lipid CD signals. Taking into consideration the susceptibility of CD to aromatic ring spatial orientation and a previous study with vibrational circular dichroism (VCD) performed on enantiomers **1**, which demonstrated that the screw‐sense preference of the peptides was not altered once embedded in the membrane,^[8]^ we concluded that the 220 nm CD sign inversion from solution to the membrane in the enantiopure peptides is induced by alteration in the relative orientation of the aromatic groups rather than structural changes in the peptide backbone.

*Linear dichroism*: The LD spectra of (*S*)‐**1**, (*R*)‐**1** and *rac*‐**1** at two different concentrations embedded in lipid vesicles were recorded (Figure 3c, d). All peptides insert into the membrane (i. e., present n‐π* signals). The LD spectra resemble those of (*S*)‐**2** and (*R*)‐**2** where data are available, though suffer more from scattering (so have a much higher wavelength cut‐off) and have less pronounced aromatic signals, which suggests cancelling orientations of the two phenyl groups. In contrast to the enantiomers **2**, foldamer (*S*)‐**1** has a slightly larger absorbance LD than (*R*)‐**1** due either to better solubilisation within the bilayer or because the enantiomers have a different geometry in the membrane, with (*R*)‐**1** being more tilted. The orientation of *rac*‐**1** is more complex; the LD data suggests that the racemic mixture binds to the membrane at both loadings (5 : 1 and 10 : 1 lipid/peptide), with the shape of the signal more similar to that of (*S*)‐**1**. The short‐axis phenylalanine transitions are positive in sign indicating that, on average, direction is parallel to the surface. The sign is mirrored by the tail of the 210 nm long axis phenylalanine transition, which is also positive.

**Figure 4:**
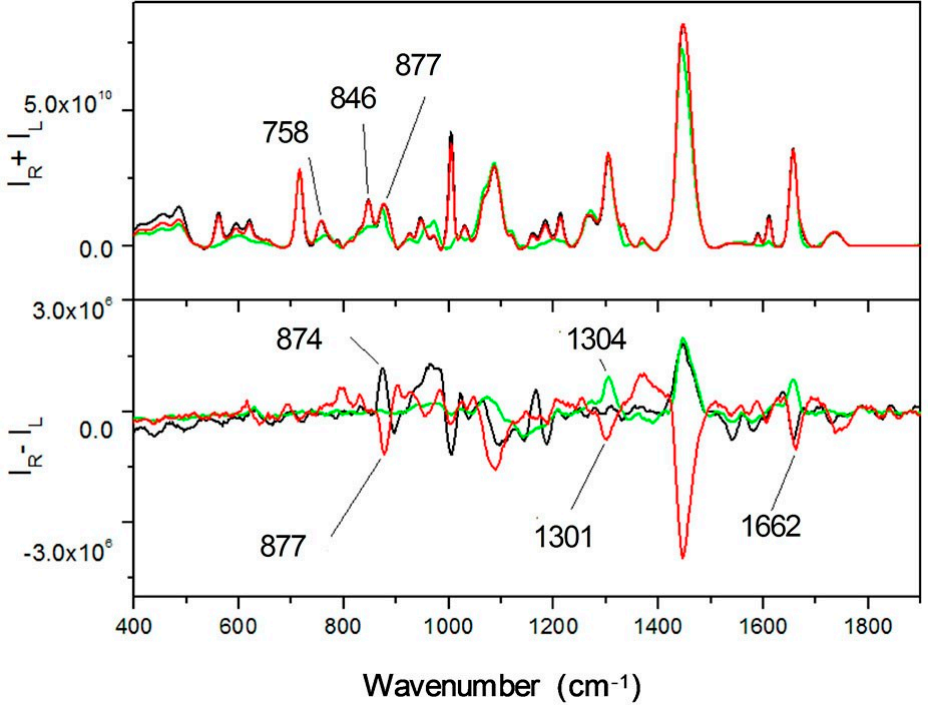
a) Raman and b) ROA of vesicle suspensions in PBS/D_2_O: DOPC vesicles (220 mg/mL lipid, **—**), DOPC vesicles doped with (*S*)‐**1** (ca. 4 : 1 lipid/peptide ratio, **—**), and DOPC vesicles doped (*R*)‐**1** (ca. 4 : 1 lipid/peptide ratio, **—**).

*Raman and ROA*: Previous ROA and VCD studies of peptides (*S*)‐**1** and (*R*)‐**1** have shown that these peptides adopt a partial 3_10_ helix conformation in organic solvents like DMSO.^[8]^ Figure 4 shows the Raman and ROA spectra of (*S*)‐**1** and (*R*)‐**1** embedded in vesicles. The DOPC used for the experiment was 100 % in the *R* configuration and therefore it can be expected to generate ROA bands. The Raman spectrum of DOPC vesicles (green line, Figure 4a) shows a peak at ∼1270 and ∼1655 cm^−1^ from the *cis* C=C stretching vibration, a strong peak at ∼1445 cm^−1^ from CH_2_ bending, a band at ∼1304 cm^−1^ from the CH_2_ twist, and a band at 1734 cm^−1^ originating from the ester vibration.^[31a]^

Raman spectra of the lipid/peptide suspensions (Figure 4a, red and black lines) are dominated by the lipid peaks, however the amide III and amide I regions show signature peaks from the peptides **1**. In particular, the Raman spectra show three peaks between 1100 cm^−1^ as and 1200 cm^−1^ as well as the peaks at 1003 cm^−1^ and 1610 cm^−1^ arising from the *υ*(CC) aromatic ring vibrations of the peptides.^[8]^ In the lower wavenumber regions there are three peaks between 559 cm^−1^ and 618 cm^−1^, which arise from the aromatic ring.[^31b^, ^31c^] Raman spectra of similar aromatic molecules such as 1‐phenylethanol and phenylalanine in aqueous solution also report a peak in this region at 620 cm^−1^. The appearance of two slightly red shifted peaks could be the result of conjugation of the aromatic or the alteration of the rotational angle of the aromatic group influenced by the lipidic environment.[^31b^, ^31c^] The band at ∼1734 cm^−1^ in the lipid/peptide suspension presents a shoulder at 1720 cm^−1^, which is similar to the Raman shift of the *tert*‐butyl ester vibration previously reported for peptide (*S*)‐**1** in [D_6_]DMSO.^[8]^

The ROA spectra of DOPC and DOPC/peptide vesicle suspensions (Figure 4b) are novel but quite complex. Due to the the lack of literature precedent it is not possible to assign all of the peaks. Although the ROA spectra appear dominated by lipid signals, peptide insertion produces changes in the ROA spectra compared to undoped vesicles. In particular, the ROA spectra of the undoped vesicles presents a positive band at 1655 cm^−1^ arising from the double bond vibration of the lipids. The amide I region of vesicles doped with (*R*)‐**1** or (*S*)‐**1** presents a peak at ∼1637 cm^−1^ (+)/1660 cm^−1^ (−), which is similar to the amide I peaks for (*S*)‐**1** in DMSO.[^31b^, ^31c^] Both peptide/lipid samples registered a sharp negative peak at 1003 cm^−1^ with the (*R*)‐**1**/DOPC mixture having a sharper peak compared to the (*S*)‐**1**/DOPC mixture; this peak is absent for undoped DOPC vesicles. Undoped vesicles present a positive peak at 1307 cm^−1^, which is absent in the spectrum of (*S*)‐**1**/DOPC and becomes negative for (*R*)‐**1**/DOPC. Lastly, there is a peak at 895 cm^−1^ that arises from the aromatic vibration, which shows a mirror image response between the two peptides and is absent in the spectum of the undoped vesicles. To interpret these ROA data, the peaks were classified into three groups: peaks that contain primarily information from the lipids, peaks that contain a mixture of information from lipids and peptides, and peaks that arise only from the peptide vibrations. We also compared with the ROA spectra of relevant peptides in solution. Peptides **1** have lower solubility in CHCl_3_ than peptides **2**, and although reproducible ROA data for **1** was obtained in [D_6_]DMSO, this solvent strongly interferes with the ROA spectra in key wavenumber ranges.^[8]^ However, the ROA spectra of peptides **2** in CHCl_3_ provide a useful comparison for ROA spectra of the doped vesicles.^[8]^

Based on published and unpublished data within our group,^[8]^ we concluded that the peaks at 1307 cm^−1^ and 1660 cm^−1^ contain information from both lipid and peptide; at this stage they are difficult to interpret, however, the difference in the signs of these peaks between doped and undoped vesicles indicates that peptide chirality influences lipid signals. The peak at 1445 cm^−1^ is dominated by lipid vibrations as the shape and sign of the peak are different from the corresponding side chain vibration of peptides **1** in [D_6_]DMSO or peptides **2** in CHCl_3_. However, the chiral response of this band, which arises mainly from the lipid tails, is influenced by the embedded peptides as there is an inversion in sign between (*S*)‐**1**/DOPC and (*R*)‐**1**/DOPC. This sign inversion is consistent with these peptides preferentially partitioning into the lipid tail region, leading to chirality transfer between the peptides and the lipids. The peaks at lower wavenumber arise only from peptide vibrations. In particular, the peak at 1000 cm^−1^ provides information on the aromatic rings. ROA studies on peptides with similar structure showed two peaks of equal intensity at 1000 cm^−1^ and 1070 cm^−1^ that arise from the aromatic ring vibration. However, for both enantiomers of **1** in bilayers, the ring breathing vibration at 1070 cm^−1^ is weaker than the peak at 1000 cm^−1^. Both enantiomers give peaks of the same sign, thus suggesting that the bilayer strongly influences these vibrations, which are the same sign as the 1000 cm^−1^ peak in the ROA spectra of (*S*)‐**2** in CHCl_3_ and (*R*)‐phenylethanol.^[31b]^ Lastly, a peak at 895 cm^−1^ appears not to be influenced by the bilayer environment and for (*S*)‐**1**/DOPC it has the same sign as (*R*)‐**2** in CHCl_3_, both of which preferentially adopt left‐handed helices in organic solvents.

### Orientation and partitioning of peptide 3 and peptide (*S*)‐4

In order to explore further how terminus modification may affect membrane insertion, we considered two peptides with a difluorinated probe and triethyleneglycol (TEG) ‘tail’ on the C terminus that improved solubility in organic solvents and membranes.^[26]^ The peptides selected were part of a larger study into conformational change in phospholipid bilayers,[^26^, ^32^] specifically mimicking light‐induced conformational changes in membrane‐bound bio‐molecules like rhodopsin.^[26]^

From the available library of difluorinated peptides, we selected peptide **3** and peptide (*S*)‐**4**. Peptide **3** contains ten residues, nine unmodified Aib residues and one difluorinated Aib residue, with a C‐terminal TEG. If this peptide folds into a 3_10_ helix, it would be about 2 nm long, similar to the width of the hydrophobic region in a typical phospholipid bilayer.^[33]^ Studies on unmodified Aib peptides with similar length showed evidence of high conductance pore‐forming structures once the peptides were embedded into lipid bilayers.[^7b^, ^17^, ^32^] Peptide (*S*)‐**4** contains the same TEG group at the C terminus; however, this peptide contains only six residues and it is too short to span the width of the membrane.

Spectroscopic comparison of these peptides in methanol solution (by solution phase ^19^F NMR) and in DOPC bilayers (by solid state ^19^F NMR) suggested that a helical peptide conformation is mostly retained from solution to the membrane, albeit with some lowering of the helical excess (*h.e*.; the excess of *P* over *M* helical conformations or vice versa) for chiral foldamers in the bilayer compared to organic solvent.^[26]^ The ^19^F ss‐NMR signal from CH_2_F groups in a shorter (Aib_5_) analogue of **3** remained unsplit in the DOPC bilayer, suggesting that the chirality of the DOPC matrix did not induce a measurable screw‐sense preference in an otherwise achiral peptide.^[26]^

**Figure 5:**
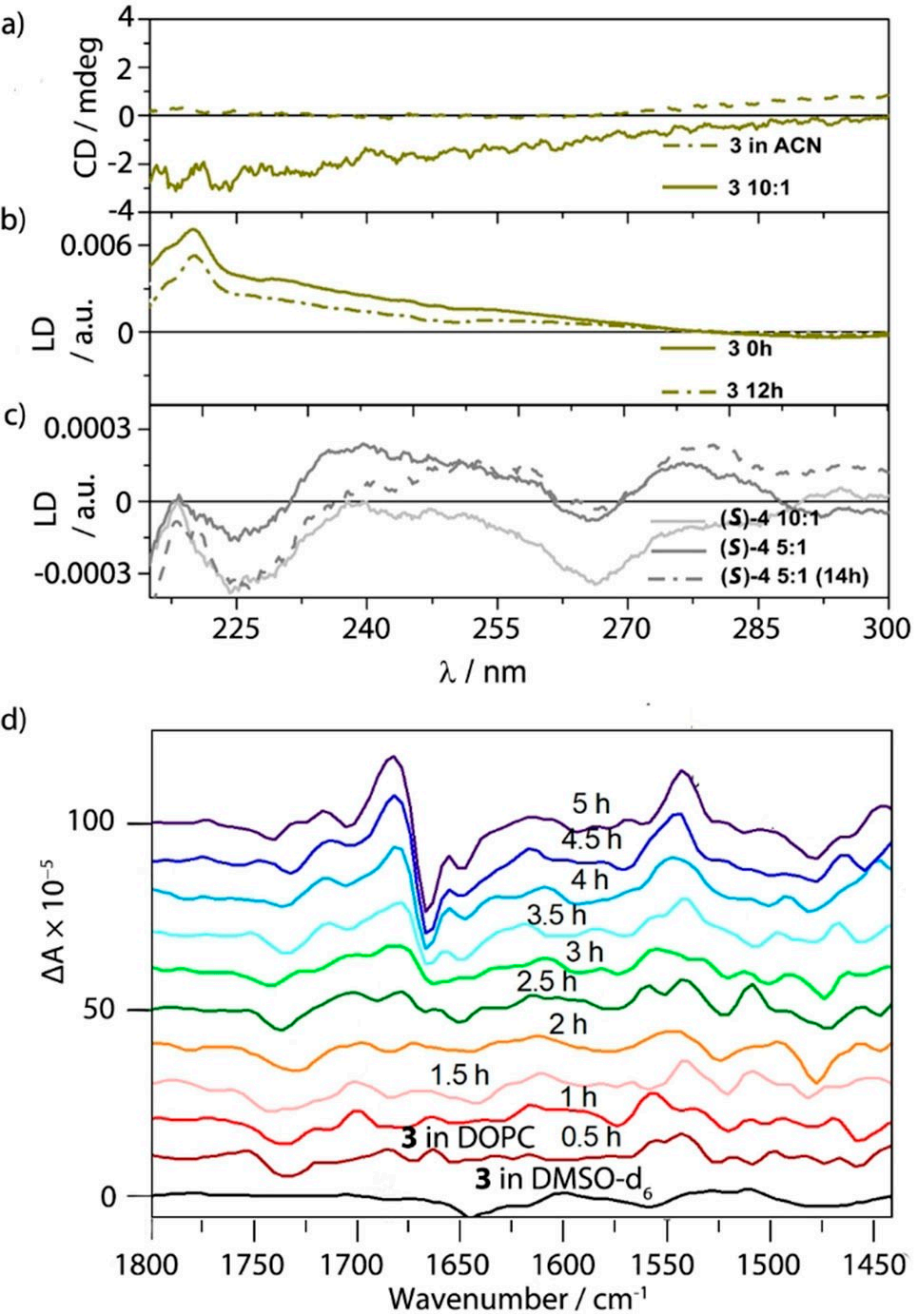
a) CD spectra of peptide **3** in acetonitrile (**‐ ‐ ‐ ‐**, 0.1 mg/mL) and in DOPC bilayers (**—**, 10 : 1 lipid/peptide ratio at 5 mg/mL lipid concentration, pH 7.4). b) LD spectra of peptide **3** at 10 : 1 lipid/peptide ratio (5 mg/mL lipid, pH 7.4) fresh (0 h, **—**) and after 12 h (**‐ ‐ ‐ ‐**). c) LD spectra of peptide (*S*)‐**4** at 5 : 1 lipid/peptide ratio, freshly prepared (**—**) and after 14 h (‐ ‐ ‐ ‐) and at 10 : 1 lipid/peptide ratio (**—**). d) VCD spectra of peptide **3** in [D_6_]DMSO (**—**) and in DOPC vesicles (ca. 4 : 1 lipid/peptide ratio) acquired over 30‐min intervals over a 5 h period.

*Circular dichroism*: Foldamer **3** is achiral, so organic solutions of this peptide do not register a CD signal, however, once the peptide is embedded within the DOPC bilayer, it gains a very small CD signal (Figure 5a). A previously published ^19^F ss‐NMR spectrum suggested that the membrane had no detectable effect on the *h.e*. of a shorter analogue of **3**.^[26]^ However, the CD spectrum of peptide **3** in the membrane presents a negative signal at about 220 nm, which by comparison with the CD spectrum of (*R*)‐**2** suggests that the peptide has a greater proportion of left‐handed helix once embedded, although the magnitude of this effect is unclear. This observation was supported by VCD investigations of peptide **3** embedded in lipid bilayers. Although during the first 30 min of a timecourse there was no discernible signal and the VCD spectrum resembles that of **3** in [D_6_]DMSO solution, later VCD spectra acquired over 5 h show the slow appearance of a signal (Figure 5d). The amplitude of the amide I’ VCD signal (shown on the top right‐hand corner in Figure 5d) did not change thereafter, suggesting that the final helical excess (*h.e*.) has been reached, and the sign of the VCD signal shows the proportion of left‐handed helix (*M* over *P*).^[8]^ We suggest that the appearance of the VCD signal is a result of an *h.e*. induced by the chiral phospholipids and speculate that this change might be related to the slow decrease observed in the LD signal. The chirality of the phospholipid matrix has previously been shown to produce diastereomeric conformations in chiral Aib‐rich peptides,^[32b]^ although the magnitude of chiral induction was unclear. Similarly, these VCD data align with the ROA studies reported herein, which showed that the left‐handed peptide (*S*)‐**1** and right handed peptide (*R*)‐**1** did not interact in the same way with the lipid bilayer. The VCD and CD studies on this achiral Aib‐rich peptide **3** suggest that there is some transfer of chirality from the lipid enviroment to the peptide, with a left‐handed helix conformation having a more favourable interaction with phospholipids in the natural *R* configuration.

*Linear dichroism*: The LD spectra of **3** reported in Figure 5b (freshly prepared and measured after 12 h incubation at room temperature) are approximately the inverse of those for both (*S*)‐**2** and (*R*)‐**2**, which is entirely consistent with the peptide inserting into and spanning the membrane. Peptide **3** orients perpendicular to the membrane surface within a few minutes of partitioning in the lipid bilayer, although the LD signal slowly weakens over time. In stark contrast, peptide (*S*)‐**4**, despite bearing the same membrane targeting C terminus has very weak LD signals (Figure 5c, freshly prepared and after 14 h of incubation at room temperature) and the challenges of baseline subtraction are very apparent in the data. Overall, either significantly less peptide (*S*)‐**4** has inserted or it adopts a tilted orientation close to the magic angle. A previous ^19^F ss‐NMR study on this peptide as well as NR experiments (discussed below), suggested that peptide (*S*)‐**4** is indeed soluble within bilayers quickly adopting its final orientation within the lipids. Based on the weak LD signal, we conclude that (S)‐**4** has a tilted insertion geometry relative to the membrane surface.

### Effect of the peptides on the membrane structure

To ascertain the effect of the Aib‐rich peptides on membrane structure, we performed neutron reflectivity (NR) experiments on supported lipid bilayers doped with: (*R*)‐**1**, (*S*)‐**1**, *rac*‐**1**, (S)‐**2**, **3** and (*S*)‐**4**. These peptides represent the different binding modes we observed with spectroscopy, namely more parallel to the membrane, more perpendicular to the membrane, and intermediate orientations. The experiments were performed on supported 1,2‐dimyristoyl‐*sn*‐glycero‐3‐phosphocholine (DMPC) and deuterated DMPC (d‐DMPC) bilayers assembled on a silicon crystal support. DMPC was chosen for its convenient transition temperature *T*_m_∼24 °C, which marks the gel–fluid phase transition,^[34]^ and its availability in a deuterated form.

**Figure 6:**
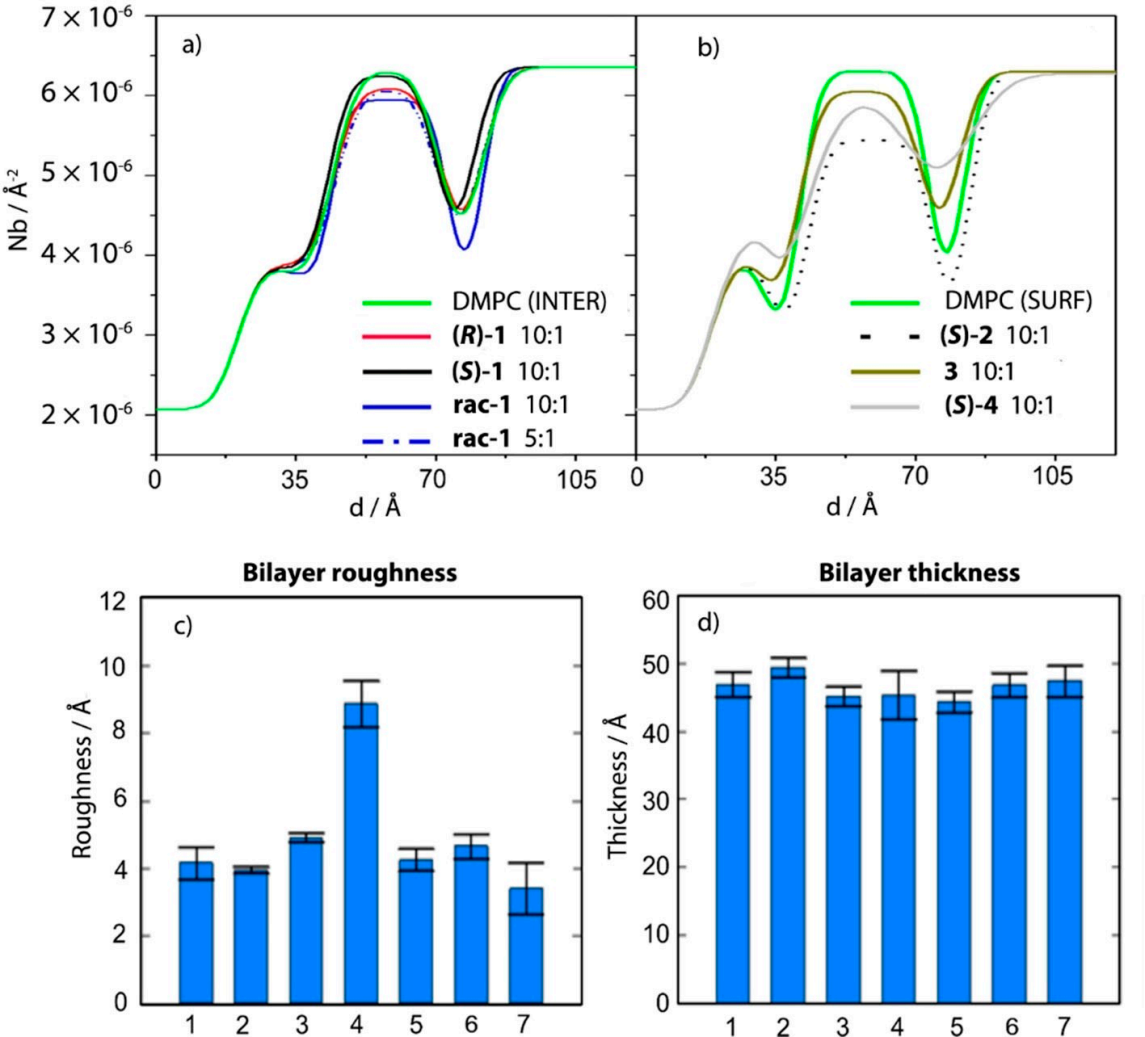
a),b) Scattering length density profiles of d‐DMPC in deuterated aqueous media (Contrast 1). a) At 37 °C in the presence of: no peptide (**—**); (*S*)‐**1** (**—**, 10 : 1 lipid/peptide ratio); (*R*)‐**1** (**—**, 10 : 1 ratio); *rac*‐**1** (**—**, 10 : 1 ratio). b) At 20 °C in the presence of: no peptide (**—**); (*S*)‐**2** (**⋅⋅⋅⋅**, 10 : 1 ratio), **3** (**—**, 10 : 1 ratio); (*S*)‐**4** (**—**, 10 : 1 ratio). Empirical values of the c) roughness and d) thickness of each d‐DMPC bilayer studied: 1: lipid only, 2: with (*S*)‐**2**, 3: with **3**, 4: with (*S*)‐**4**, 5: with (*R*)‐**1**, 6: with (*S*)‐**1**, 7: with *rac*‐**1**. All with lipid/peptide ratio 10 : 1.

The neutron reflectivity measurements were performed in two separate experiments. The experiments for peptides (*S*)‐**4**, **3** and **2** were performed on SURF with the experiment carried out at room temperature of 20±1 °C. The experiments for (*S*)‐**1**, (*R*)‐**1** and *rac*‐**1** were performed on INTER with a temperature maintained at 37±1 °C by means of a circulating water bath. The difference in temperature puts the h/d‐DMPC bilayer in the gel phase for the SURF experiment and in the fluid phase for the INTER experiment. For this reason, direct comparisons between the two experiments may not be appropriate and the two are discussed separately. For both experiments the structure of the h/d‐DMPC bilayer alone was measured first and used as a reference to monitor the changes in the properties of the membrane. Overall, the data were analysed by comparing the scattering density profiles of the bilayer with the scattering profile of the lipid/peptide suspensions (Figure 6a, b). The parameters of the undoped vesicles can be found in Supporting Information.

*Peptides in fluid phase lipid bilayers*: The neutron reflectivity experiments for (*R*)‐**1**, (*S*)‐**1** and *rac*‐**1** in fluid phase lipid bilayers were performed at both 5 : 1 and 10 : 1 lipid/peptide ratios. The h‐DMPC bilayers in D_2_O for the lipid/(*S*)‐**1** sample (10 : 1 lipid/peptide ratio) and lipid/*rac*‐**1** (10 : 1 ratio) reported a Bragg peak at Q∼0.103 Å^−1^ (Figure S1–S10 in the Supporting Information), suggesting the presence of patches of multilayered lipids (additional details can be found in Supporting Information). Overall, the bilayer thickness in the presence of the peptides did not change noticeably compared to the bare lipids, with all results within 4 % (Figure 6d). Comparison with undoped bilayers showed **1** and *rac*‐**1** did not induce major changes in either bilayer thickness or roughness (bars 5–7 in Figure 6c,d), a clear indication that the peptides are fully embedded within the lipid with little or no protrusion towards the bulk phase. The scattering length density profiles for the deuterated bilayers in D_2_O (Contrast 1) are the most sensitive to variation so we focused our data interpretation on this contrast (Figure 6a, see the Supporting Information for uncorrected profiles and the scattering length density profiles for all other contrasts). Overall, irrespective of membrane loading, the peptides induce little alteration in the roughness and thickness of the membrane.

*Peptides in gel phase lipid bilayers*: Peptides (*S*)‐**2**, (*R*)‐**2**, (*S*)‐**4** and the achiral peptide **3** were tested with the lipid bilayer in the gel phase. The DMPC scattering length density profiles after addition of the peptides showed very little change in the overall layer thickness, with values within 5 % of that for the bare lipid bilayer (Figure 6d).[^2^, ^3^, ^4^] Both enantiomers of **2** preferentially localize in the tail‐group region Peptides **3** and (*S*)‐**4** also preferentially partitioned into the tail‐group region, albeit to a lesser extent. The scattering profiles for peptides **2** and **3** are very similar to that of the undoped bilayer (Figure 6b), which implies that the peptides do not induce fundamental structural alterations within the supported bilayer. In contrast, peptide (*S*)‐**4** causes the bilayer roughness to more than double in value, shifting from 4 to 9 Å. The sharp increase in this value suggests that locating (*S*)‐**4** within the bilayer causes the lipid head‐group and tail to interweave and the separation between the different regions is blurred (bar 4 in Figure 6c).

Overall, peptides **1**, **2** and **3** interact strongly with the membranes, as observed by CD and LD, yet produced very little alteration of the membrane structure. This model is consistent with the LD data (as well as reported NMR^[26]^ and HPTS^[9]^ studies) showing peptides of similar sequence and length embed into the membranes.

Interestingly, peptide (*S*)‐**4** had a greater effect on the supported membrane. Like **3**, this peptide has a TEG group at the C terminus, however unlike **3** it is too short to cross the hydrophobic region of the bilayer. NR studies on peptides of similar length and sequence suggest that Aib‐rich peptides induce localized thinning of the bilayer.^[5]^ In this study, peptide (*S*)‐**4** does affect the membrane structure but it induces a sharp increase of the roughness rather than thinning of the bilayer. We speculate that high peptide loadings produce large clusters of peptide in close proximity, which induces an average thinning of the bilayer. If the peptides are less abundant in the bilayer, this may perhaps lead to regions of thinning coexisting with regions where the bilayer structure is little affected. The net result is an undulation of the surface and the increase in roughness observed during our experiments. The reason for this change being so pronounced for (*S*)‐**4** is unclear at this stage.

### Molecular dynamics simulations

**Figure 7:**
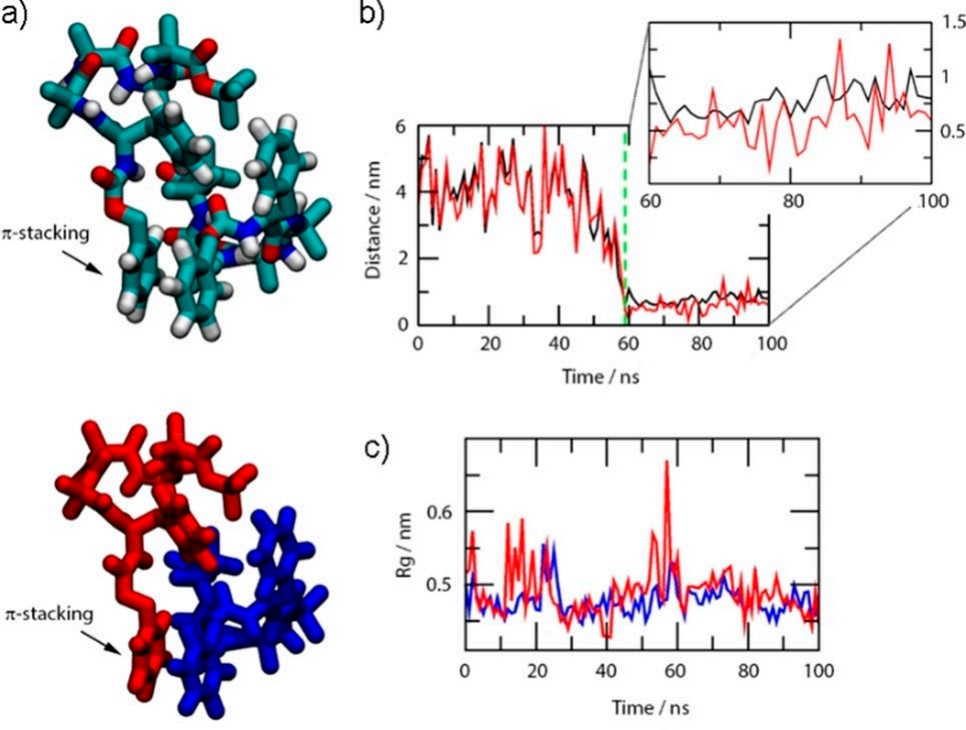
a) Snapshots of the peptide dimer formed in a bulk solution simulation (water molecules are omitted for clarity). Top: Atoms are coloured red: oxygen, white: hydrogen, blue: nitrogen and cyan: carbon. Bottom: Individual peptides are red or blue. b) Corresponding time series for the distance between the two peptides (**—**) and their aromatic rings (**—**) based on centre‐of‐mass positions. The dashed green line represents the time when the dimer is formed (based on a 1 nm cut‐off distance). c) Time series for the radius of gyration of the peptides that formed the dimer.

Molecular dynamics (MD) simulations were performed for (*S*)‐**1**, which had the most resolved LD spectrum, to ascertain the orientation adopted by this short peptide in a bilayer membrane. The peptide was first simulated in bulk water, which showed (*S*)‐**1** folded into left‐handed helices (as favoured by an l‐Phe cap^[29]^) that clustered into stable dimers with π‐stacking interactions between the aromatic rings (Figure 7) and attractive interactions between the peptide backbones. The average radius of gyration was 0.5 nm and 0.5 nm for the molecules that formed the (*S*)‐**1** dimer. Each monomer had an average end‐to‐end length of 0.9 nm and 1.0 nm.

Either monomeric or dimeric peptides were then placed above DOPC membranes, surrounded by water and the simulations run for 500 ns. Contact analyses (data not shown) indicated that peptides reached equilibrium positions in the bilayer (data are converged) in the monomer simulation, whereas convergence is less clear for the dimer simulation. The contact analysis once the monomer had penetrated into the bilayer showed that the majority (>2/3) of the peptide–lipid interactions involve acyl chains and not the head groups.

**Figure 8:**
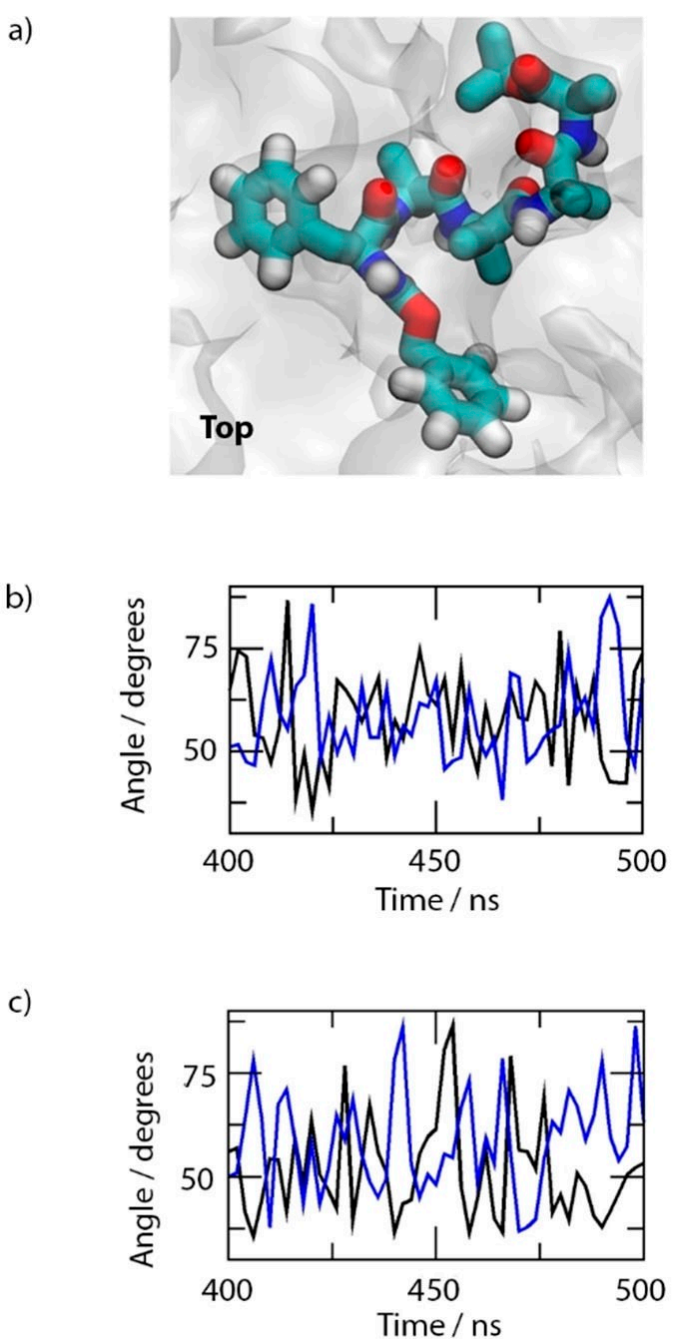
a) Final frame, top‐view snapshot of the (*S*)‐**1** monomer embedded in the DOPC bilayer (water molecules are omitted for clarity). Atoms are coloured red: oxygen, white: hydrogen, blue: nitrogen and cyan: carbon. The DOPC bilayer is shown as a translucent white surface. Time series for the long (**—**) and short axis (**—**) angles for the b) mid‐chain and c) terminal phenyl rings relative to the membrane normal.

The backbone orientation of the monomer in the bilayer is clearly more parallel than perpendicular to the membrane surface (Figure 8a, top view). This means that the 220 nm n–π* transition, which is polarized perpendicular to the helix backbone, is expected to give a negative signal in accord with the LD experiment. The 210 nm peptide signal is conversely expected to be positive, as we observed (see the Supporting Information). However, it should be noted that the phenyl groups have a long‐axis polarized transition also at this wavelength.

**Figure 9:**
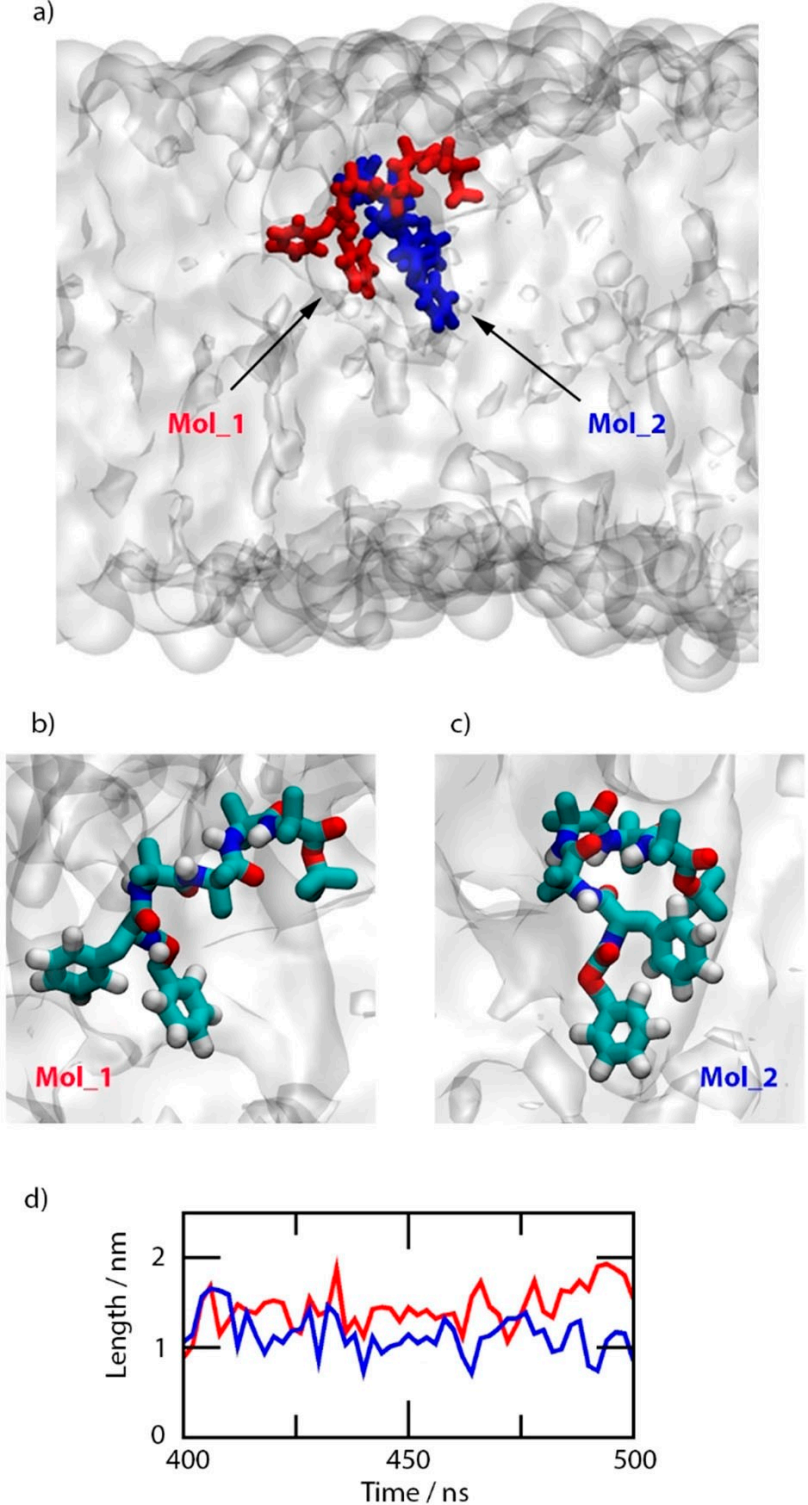
a) Final frame, side view snapshot of the (*S*)‐**1** dimer embedded in the DOPC lipid bilayer. The molecules that form the (*S*)‐**1** dimer are red (Mol_1) and blue (Mol_2). The DOPC bilayer surface is shown as a translucent white surface, and the water molecules are omitted. b) Side view perspective of the first (*S*)‐**1** molecule, i. e., Mol_1; c) Side view perspective of the other (*S*)‐**1** molecule, i. e. Mol_2. The atoms are coloured red: oxygen, white: hydrogen, blue: nitrogen and cyan: carbon. The DOPC bilayer surface is shown as a translucent white surface and the water molecules are omitted. d) End‐to‐end length for the two (*S*)‐**1** peptides (colours as in (a)) during the last 100 ns of simulation time.

During the bilayer simulations with two peptides per simulation cell, (*S*)‐**1** initially clustered into dimers in the aqueous phase. As the peptide dimers passed through the water‐lipid interface the orientation of the aromatic groups changed to accommodate a combination of π‐stacking interactions between the (*S*)‐**1** monomers and interactions of the peptide aromatic rings with the DOPC lipid tails (Figure 9). The average radii of gyration in the lipids were marginally larger than in aqueous phase and the average lipidic dimer end‐to‐end lengths (of a more linear structure than the aqueous one) were 1.2 nm and 1.5 nm during the last 100 ns. Although the two peptides of the dimer orient differently, they also have the same signs for the peptide transition LD signal as found for the monomer in the bilayer.

The orientations of the benzyl groups are less obvious from Figures 8a and 9, though it is clear that the peptide backbones preclude them from slotting parallel to the lipids, as is commonly observed for aromatic molecules.^[37]^ The angles made by the long and short axes of the benzyl groups to the membrane normal were therefore computed for lipidic (*S*)‐**1** monomer and dimer simulations from the last 100/50 ns of the simulations. The two monomer phenyl short axis transitions appear at 270 nm and have average orientations in the final 100 ns of the lipidic monomer simulation of 53° (terminal) and 58° (mid‐chain) which is in accord with a small net positive signal (the zero point is at 54.7°) at 270 nm. In this case the aromatic groups are close to the magic angle, in accord with the small LD signals observed despite the high concentrations. Were the dimer to be present, the simulations present a very slightly negative LD signal at 270 nm (angles of 47°, 53°, 53° and 54°) which is not in accord with the experimental results. The 210 nm phenyl LD signals are small and negative for the monomer and very small and negative for the dimer. So, we conclude that the peptide signal dominates here. Overall, we conclude that the peptide dimers dissociate upon insertion into the membrane and the peptides lie more‐or‐less parallel to the surface with the phenyl groups lying tilted at just more than the magic angle.

Bilayer properties were calculated for all peptide‐lipid simulations to quantify bilayer deformation by the peptide. The bilayer thickness and area per lipid changed by no more than 1 % for both monomer and dimer peptide simulations. The order parameter, which quantifies the angle between lipid chain bonds and the bilayer normal, indicates, however, that the peptides affect lipid tail orientation (data not shown). These results are in accord with the LD orientation and lipid‐interaction data from our Raman and ROA spectra.

## Conclusion

Combining data derived from five different spectroscopic techniques, molecular dynamics and neutron reflectivity has provided a better understanding of how Aib‐rich peptides with a terminal aromatic group interact with lipid bilayers. For our peptides of interest, **1** (both enantiomers) as well as closely related **2**, CD, LD and NR experiments, supported by MD simulations, lead us to conclude that each enantiomer inserts easily into the membrane, with these short peptides adopting a helical conformation in the membrane that has the helix axis oriented approximately parallel to the bilayer surface. The MD simulations also suggest that peptide interactions with the lipids are at least as energetically favourable as the peptide–peptide interactions proposed to cause peptide **1** to dimerise in an aqueous medium. Other than local perturbations, the simulations show that the membrane accommodates the peptides without overall alteration of its structure, which is in accord with NR data at membrane loadings of 10 : 1 lipid/peptide ratio and the poor antibacterial activity of similar short peptides against *Bacillus megaterium*.^[5]^

Achiral peptide **3** with a C‐terminal TEG group is similar to the short peptides, but is long enough to span the hydrophobic width of the membrane. LD shows that the favoured orientation of this peptide is perpendicular to the bilayer, which it adopts without significantly perturbing the bilayer structure. This is in contrast with the short peptide (*S*)‐**4**, also bearing a C‐terminal TEG, which is the only one of the peptides studied here that NR showed measurably altered membrane structure. However, the alteration induced by (*S*)‐**4** at the concentration studied was not sufficient to disrupt the bilayer.

Despite the chirality of the lipids potentially producing diastereomeric conformations in the enantiomers of peptide **1**, the difference between the binding modes of the enantiomers of **1** with the membrane is small, although consistently observed with both LD and NR experiments. The small difference observed is perhaps because the peptides preferentially locate in the tail group region and, therefore, are sufficiently distant from the lipidic chiral centres of the head group region. This interpretation is supported by the ROA measurements of DOPC vesicles doped with either (*S*)‐**1** or (*R*)‐**1**, which showed a clear influence of these chiral peptides on the ROA signal from the double bond in the acyl tails of DOPC. In other work, enantiomers of short chiral Aib foldamers bearing fluorescent labels have been shown to adopt different conformations in egg yolk phosphatidylcholine bilayers, confirming that an interplay exists between phospholipid chirality and peptide conformation.^[32]^ Moreover, CD measurements and a time‐course VCD experiment on peptide **3** showed in bilayers composed of natural *R* phospholipids, this achiral peptide slowly showed a preference for a left‐handed helical conformation although the helical excess is unknown. Chiral interactions between the bilayer and chiral and achiral peptides are clear from these and other studies, but the magnitude of this effect remains unclear in general and may depend upon peptide identity, location and orientation in the membrane.

Although the chirality of the lipid bilayer is often overlooked, in recent years it has become more evident that the chirality of the membrane plays a role in molecular recognition and in the kinetics of distribution.[^1e^, ^1f^, ^1g^] Therefore, in order to understand the relationship between antimicrobial activity and peptide sequence and to move forward the design of antimicrobial peptides, it is important to consider the chirality of both the peptides and the constituents of the target membrane.

## Experimental Section

**Materials**: All peptides were synthesized according to previously published procedures.[^18^, ^24a^] The lipid used for the Raman, ROA, LD, VCD and CD experiments was DOPC (1,2‐dioleoyl‐*sn*‐glycero‐3‐phosphocholine). For the Neutron Reflectivity (NR) experiments, DMPC (1,2‐dimyristoyl‐*sn*‐glycero‐3‐phosphocholine) and tail‐deuterated DMPC (d‐DMPC, 1,2‐dimyristoyl‐D_54_‐*sn*‐glycero‐3‐phosphocholine) were used. All lipids were purchased from Avanti Polar Lipids (Alabaster, AL, USA). The buffer used for the ROA, LD and CD experiments was PBS (0.01 M, sodium chloride concentration of 0.154 M, pH 7.4, made up in D_2_O or H_2_O as appropriate), obtained from Sigma–Aldrich. NR experiments were performed using HEPES buffer in D_2_O (10 mM) with NaCl (150 mM) and CaCl_2_ (2 mM; all reagents from Sigma–Aldrich).

**Instrumentation**: The LD and CD experiments were performed on a Jasco J‐815 spectropolarimeter adapted for LD experiments. NR measurements were carried out using the SURF^[38]^ and INTER^[39]^ time‐of‐flight reflectometers at the Rutherford Appleton Laboratory (Oxfordshire, UK). Raman and ROA experiments were performed using a ChiralRaman2x (BioTools Inc., USA) operating at 532 nm excitation. The IR and VCD spectra were acquired with the Chiral IR‐2X VCD spectrometer (BioTools, Inc., USA).

**Methods**: Small unilamellar vesicles (SUVs) for Raman, ROA, LD and CD experiments were prepared by drying, first under reduced pressure and then under high vacuum for 2 h, a chloroform solution of lipids onto the walls of a round‐bottomed flask. The film was then re‐suspended in PBS buffer (pH 7.4, H_2_O or D_2_O as required) to obtain a turbid suspension. To perform LD and CD experiments, the lipids underwent 5 cycles of freeze/thaw and were then transferred into the 1 mL gastight syringe of a LiposoFast extruder. The extruder was fitted with a 100 nm pore polycarbonate membrane and the lipid suspension was passed through the membrane at least 15 times on the day of the experiment. The size of the vesicles was checked with dynamic light scattering (DLS) as soon as they were formed, and the maintenance of their size was also confirmed after each CD and LD experiment.

To perform Raman and ROA experiments, the lipids were sonicated at room temperature with a sonicator bath, the experiment were performed using 1.0 W laser power at the source. Data collection varies as appropriate (roughly ∼48 h).

The IR and VCD measurements were performed upon the DOPC and foldamer‐DOPC suspensions prepared in D_2_O‐based PBS buffer as described above for the ROA experiments. Concentrations of 220 mg mL^−1^ of DOPC and 50 mg mL^−1^ of the peptide were used to keep the lipid/foldamer weight ratio in the final mixture at about 4 : 1. The samples were placed in a dismountable BaF_2_ VCD cell with a pathlength of 50 μm. The differences in the pathlength of the dismountable VCD cell, and/or small variations in the sample concentration can produce variations in sample absorbance. The IR and VCD spectra were acquired at 8 cm^−1^ resolution for 18 h. To avoid cell and baseline artefacts, solvent spectra were measured in the same cell under the same conditions as the samples and subtracted from the sample spectra, after which baseline correction was performed. Spectra of foldamer solutions were measured once per sample. Spectra of foldamer‐DOPC suspensions were measured over several hours in 30 min slots.

The LD and CD experiments were performed by adding a small amount of the peptide solution in acetonitrile (ACN) to a suspension of small unilamellar vesicles (SUVs, 5 mg/mL) to achieve a stoichiometric ratio of 10 : 1 or 5 : 1 lipid/peptide. The peptide enantiomers and the racemic mixtures in DOPC liposomes were aligned using shear flow to obtain LD spectra in a custom LD cell built by Crystal Precision Optics (Rugby, UK).[^40^, ^41^] LD experiments were performed following methods previously published.^[42]^ CD spectra were collected using either a standard 1 mm rectangular quartz cuvette or a LD cell on the same sample used for LD data acquisition. Peptides for CD and LD (0.1 mg/mL) were measured in either acetonitrile or in a suspension of lipid at lipid/peptide ratio 10 : 1. The CD spectral range was from 180 nm to 300 nm, with a speed of 100 nm/min, an integration time of 1 second, and averaged over 8 measurements. The LD experiments were performed by transferring ∼80 μL of the lipid/peptide mixture into the LD cuvette with a rotation speed of 3000 rpm, and the baseline was measured on the same sample at 0 rpm. The LD spectral range was from 180 nm to 350 nm, with a speed of 100 nm/min, an integration time of 1 s, and each spectrum was the average of 8 measurements. The CD spectra of blank lipid vesicles or PBS solvent were measured and subtracted from the final spectra. Before and after addition of the peptides, DLS experiments were performed to check the integrity of the vesicles (data not shown).

Small unilamellar vesicles (SUVs) for the NR experiments were prepared by drying a chloroform solution of the appropriate lipids with the appropriate amount of peptide onto the walls of a round‐bottomed flask using a nitrogen flow. The film was further dried under reduced pressure overnight. The film was then re‐suspended in HEPES buffer solution using a sonicator to obtain a turbid solution. A lipid/peptide suspension was injected into a custom‐made cell which contains a single silicon crystal (SiO_2_) previously cleaned using piranha solution. The vesicles were allowed to burst and deposit onto the hydrophobic surface of the cell to form a bilayer. The excess of the suspension was removed using an HPLC pump attached to the cell. All bilayer deposition procedures were performed under ambient conditions and without sub‐phase buffering before the NR experiment.

The reflectivity was measured as a function of the momentum transfer, *Q_z_*.2

(2) $$Q{}_{z}=\frac{4\pi }{\lambda }\sin\theta$$

with *λ* representing the wavelength and *θ* the incident angle. As the instruments use different wavelength ranges (SURF 0.5–6.9 Å, INTER 1.5–17.5 Å), different angles were used to obtain full reflectivity profiles. The collimated neutron beam was reflected from the silicon–liquid interface with grazing incident angle *θ=*0.35°, 0.65° and 1.5° for SURF and *θ*=0.7° and 2.3° for INTER, the individual runs were then customarily overlapped and stitched. The flow cell was connected to a liquid chromatography pump (L7100 HPLC pump, Merck, Hitachi). For each isotopic contrast experiment, a total of 22.5 mL of 20 mM pH 7.0 (pD 7.4) sodium phosphate buffer solution, was pumped through the flow cell at a speed of 1.5 mL/min. Peptides (*S*)‐**1** and (*R*)‐**1** were measured on INTER where the temperature was maintained at 37±1 °C by means of a circulating water bath. Peptides (*S*)‐**2**, (*R*)‐**2**, **3** and **4** were measured on SURF at room temperature, 20±1 °C.

To increase the reflectivity contrast, the h‐ and d‐bilayers were measured under three different water contrasts: D_2_O, H_2_O and contrast matched silicon water (CMSi, 38 % D_2_O in H_2_O). The scheme of contrasts is shown in Supporting Information Figure 1. All data were analysed globally. For each sample measured, the fitted reflectivity profiles, the corresponding scattering length density profiles, fitting parameters and the confidence interval are shown in the Supporting Information (Figures S2–S10 and Tables S1–S9).

**Data analysis**: Raman and ROA spectra were processed using MATLAB 2018 and an in‐house toolbox. Conventional Raman spectra were baseline‐corrected according to the method proposed by Eilers et al.^[43]^ The ROA spectra were baseline corrected using a median filter, and smoothed using a second level Savitzky‐Golay filter. LD and CD data were processed using the JASCO SpectraManager 2.

Neutron reflectivity data were analysed using RasCAL (developed at ISIS Spallation Neutron Source, Rutherford Appleton Laboratory), which uses the optical matrix method to fit Abeles layers to the interfacial structure.^[38]^ The interfacial region is divided in a finite number of layers, each layer is characterised by a thickness (*d*), roughness (*σ*) and scattering length density (*ρ*). The scattering length density of the layer is the sum of the scattering length density of all components within the layer and is a function of the layer composition:

(3) $$N\rho {}_{\mathrm{layer}}=\underset{i}{\sum }N\rho {}_{i}\varphi {}_{i}$$

where *ϕ_i_* is the volume fraction of every component in the layer and all *ρ* values are given in the Supporting Information. The reflectivity is then calculated from the model and compared to the experimental data. The fitting routine is iterated to achieve a least‐square minimisation. In all cases the simplest model was selected, that which involves the minimum number of layers to describe the interfacial region. The interfacial structure was divided into four layers: silicon oxide, inner head‐group, tail‐group and outer head‐group region. The presence of a layer representing peptide protruding towards the bulk aqueous phase was also tested, but the fit invariably gave negligible thickness to this layer. In all cases the bilayers were perfectly symmetrical, that is, in all samples the inner and outer head‐group regions were identical: no measurable improvement could be achieved by introducing asymmetry within the bilayers or adding a further layer extending toward the bulk phase.[^35^, ^36^] The interfacial roughness σ was simulated using a Gaussian distribution and played a key role in the analysis. The bilayer coverage for each bilayer was determined from the hydration of the tail‐group region, which would contain no water (0 % hydration) in the event of full coverage. During the data fitting of the experiments the coverage of d‐ and h‐lipids was allowed to vary to account for differences in deposition; all other parameters were kept constant between all contrasts. The uncertainty in the fitting parameters was obtained using Bayesian probability routines available with RasCAL.

**Molecular dynamics trajectory calculations and analysis**: Simulations were performed with the GROMACS simulation package (version 5.1.2) and the GROMOS 53 A6 force field. Temperature was controlled with the Nosé–Hoover thermostat (time constant of 0.5 ps) and the pressure was maintained at 1 bar with the Parrinello‐Rhaman barostat (time constant of 2 ps). Electrostatic interactions were treated with the smooth particle mesh Ewald algorithm with a short‐range cut‐off of 1.2 nm. Van der Waals interactions were truncated at 1.2 nm and a long‐range dispersion correction was applied for energy and pressure. Molecular bonds were constrained with the LINCS algorithm, enabling the use of a 2 fs time step throughout. Average angles were calculated by defining a ‘bond’ across the short and long axes of the benzyl groups and determining their angles with an average membrane surface. The simulations were performed with one or two peptides per simulation cell and 128 molecules of either DOPC or DMPC.

## Conflict of interest

The authors declare no conflict of interest.

## Supporting information

As a service to our authors and readers, this journal provides supporting information supplied by the authors. Such materials are peer reviewed and may be re‐organized for online delivery, but are not copy‐edited or typeset. Technical support issues arising from supporting information (other than missing files) should be addressed to the authors.

Supplementary
