# Supplementary material for: Insight into the Mechanism of Action and Peptide‐Membrane Interactions of Aib‐Rich Peptides: Multitechnique Experimental and Theoretical Analysis
Source: Chembiochem. 2021 Feb 24;22(9):1656–67. doi: 10.1002/cbic.202000834 (PMC8248331; doi:10.1002/cbic.202000834)
Supplement: Supplementary file 1 — Supplementary [file CBIC-22-1656-s001.pdf]

ChemBioChem

Supporting Information

Insight into the Mechanism of Action and Peptide-Membrane Interactions of Aib-Rich Peptides: Multitechnique Experimental and Theoretical Analysis

Maria Giovanna Lizio,* Mario Campana, Matteo De Poli, Damien F. Jefferies, William Cullen, Valery Andrushchenko, Nikola P. Chmel, Petr Bouř, Syma Khalid, Jonathan Clayden, Ewan Blanch, Alison Rodger,* and Simon J. Webb

Table of Contents:

S.1 Linear Dichroism and Circular Dichroism ………………………………………………………… 2

S.1.1 Supplementary Figures……………………………………………………………….... 2

S.2 Neutron Reflectometry…………………………………………………………………………… 4

S.2.1. Tables with fitting parameters for **1**, **2**, **3**, **(*S*)-4**……………………………………………… 4

S.2.2 Supplementary Figures………………………………………………………………… 9

S.4 Molecular dynamics simulations……………………………………………………………… 18

S.4.1 Supplementary Table…...……………………………………………………………….. 18

S.4.2 Supplementary Figures………………………………………………………………… 19

S.1 Linear Dichroism and Circular Dichroism

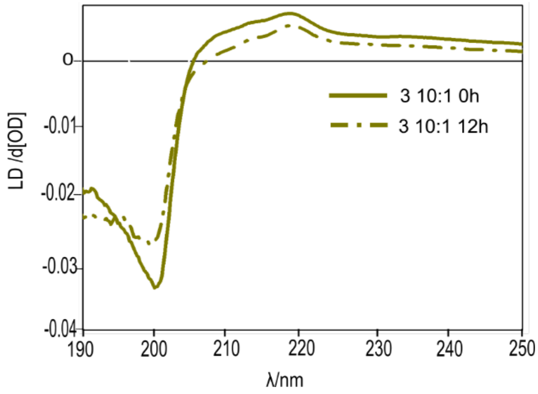

Supplementary Figure 1: LD spectra of peptide **3** at 10:1 lipid/peptide ratio (5 mg/mL lipid, pH = 7.4) fresh (0 h, solid olive line) and after 12 h (dashed olive line).

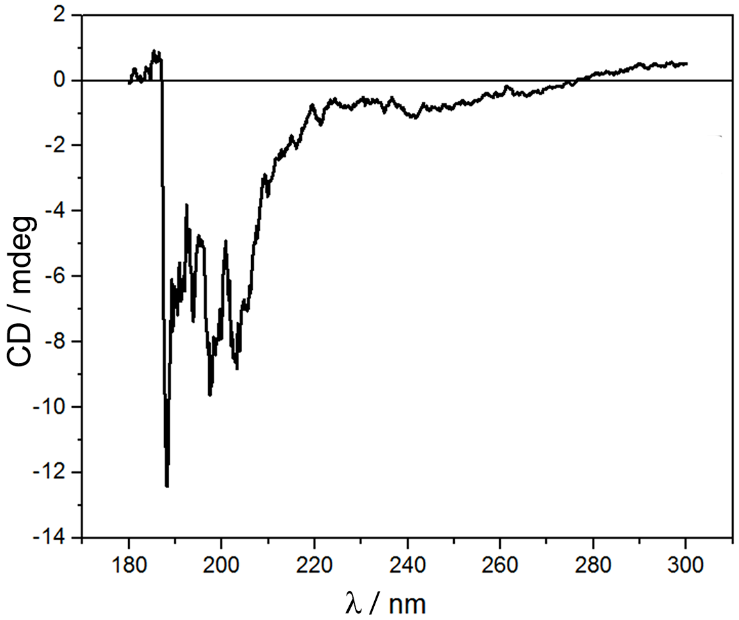

Supplementary Figure 2: CD spectrum of DOPC vesicles (5 mg/mL lipid) in PBS.

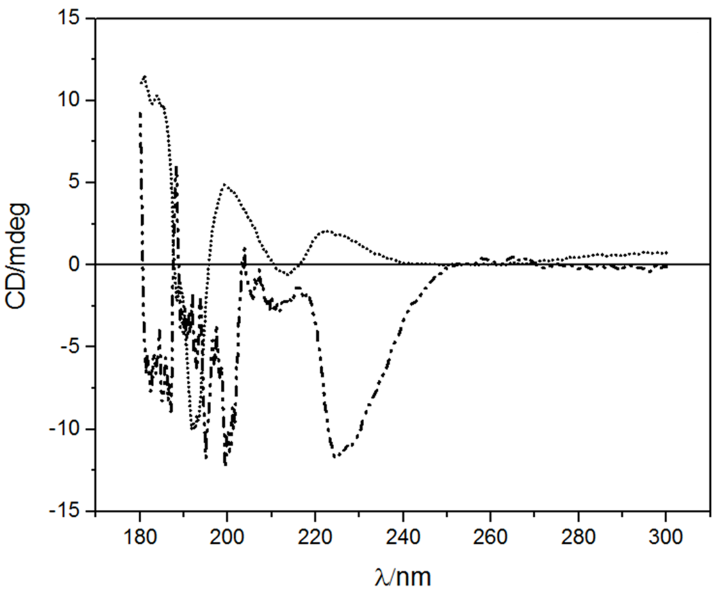

Supplementary Figure 3: CD of **(*S*)-1** in acetonitrile at 0.1 mg/mL (dotted black line) and in DOPC vesicles at a 5:1 lipid:peptide ratio (5 mg/mL lipid concentration, dashed black line).

S.2 Neutron Reflectometry

Supplementary Table 1: Fitting parameters for the DMPC bilayer measured on the reflectometer SURF at 20°C. The 95% confidence intervals obtained from the error analysis is shown in brackets.

Fitting parameter	Fitted value
Substrate roughness / Å	4.72 (4.02, 4.99)
Silicon oxide thickness / Å	15.56 (14.91, 15.98)
Silicon oxide roughness / Å	3.89 (3.03, 5.41)
Silicon oxide hydration / %	18.58 (16.37, 20.61)
Head-group thickness / Å	6.45 (5.72, 7.12)
d-Head-group hydration / %	20.08 (12.23, 26.08)
Bilayer roughness / Å	4.16 (3.63, 4.59)
Tail-group thickness / Å	33.97 (33.55, 34.42)
h-Tail-group hydration / %	0.52 (0.02, 1.57)
d-Tail-group hydration / %	12.07 (10.87, 13.42)

Supplementary Table 2: Fitting parameters for the DMPC bilayer in presence of peptide **2** measured on the reflectometer SURF at 20°C. The 95% confidence intervals obtained from the error analysis is shown in brackets.

Fitting parameter	Fitted value
Substrate roughness / Å	3.08 (3.00, 3.25)
Silicon oxide thickness / Å	10.89 (10.15, 11.59)
Silicon oxide roughness / Å	3.59 (3.01, 4.60)
Silicon oxide hydration / %	16.33 (13.29, 19.74)
Head-group thickness / Å	9.02 (8.60, 9.42)
d-Head-group hydration / %	24.45 (21.78, 27.00)
Bilayer roughness / Å	3.95 (3.90, 4.09)
Tail-group thickness / Å	31.34 (30.73, 31.94)
h-Tail-group hydration / %	0.03 (0.00, 0.11)
d-Tail-group hydration / %	1.21 (0.32, 2.13)
Peptide fraction in head-group	0.19 (0.17, 0.24)
Peptide fraction in tail-group	0.19 (0.18, 0.21)

Supplementary Table 3: Fitting parameters for the DMPC bilayer in presence of **3** measured on the reflectometer SURF at 20°C. The 95% confidence intervals obtained from the error analysis is shown in brackets.

**Fitting parameter**	**Fitted value**
Substrate roughness / Å	4.76 (4.30, 4.99)
Silicon oxide thickness / Å	11.29 (10.75, 11.99)
Silicon oxide roughness / Å	4.01 (3.05, 5.62)
Silicon oxide hydration / %	14.78 (12.46, 17.76)
Head-group thickness / Å	5.77 (5.32, 6.20)
d-Head-group hydration / %	27.77 (22.93, 29.92)
Bilayer roughness / Å	4.91 (4.70, 5.00)
Tail-group thickness / Å	33.55 (32.99, 34.05)
h-Tail-group hydration / %	6.99 (7.84, 8.63)
d-Tail-group hydration / %	11.08 (10.17, 12.11)
Peptide fraction in head-group	0.18 (0.15, 0.23)
Peptide fraction in tail-group	0.06 (0.04, 0.07)

Supplementary Table 4: Fitting parameters for the DMPC bilayer in presence of **(*S*)-4** measured on the reflectometer SURF at 20°C. The 95% confidence intervals obtained from the error analysis is shown in brackets.

**Fitting parameter**	**Fitted value**
Substrate roughness / Å	4.84 (4.44, 5.00)
Silicon oxide thickness / Å	10.83 (10.21, 11.49)
Silicon oxide roughness / Å	4.26 (3.09, 5.75)
Silicon oxide hydration / %	27.45 (24.24, 29.87)
Head-group thickness / Å	5.98 (5.18, 6.96)
d-Head-group hydration / %	13.33 (10.18, 18.65)
Bilayer roughness / Å	8.86 (8.21, 9.56)
Tail-group thickness / Å	33.33 (31.37, 34.86)
h-Tail-group hydration / %	4.77 (2.20, 6.88)
d-Tail-group hydration / %	7.03 (4.52, 9.05)
Peptide fraction in head-group	0.18 (0.15, 0.20)
Peptide fraction in tail-group	0.06 (0.02, 0.01)

Supplementary Table 5: Fitting parameters for the DMPC bilayer measured on the reflectometer INTER at 37°C. The 95% confidence intervals obtained from the error analysis is shown in brackets.

Fitting parameter	Fitted value
Substrate roughness / Å	4.80 (4.33, 4.99)
Silicon oxide thickness / Å	14.22 (13.78, 14.68)
Silicon oxide roughness / Å	5.89 (5.65, 6.00)
Silicon oxide hydration / %	16.52 (14.78, 17.99)
Head-group thickness / Å	9.41 (8.95, 9.83)
d-Head-group hydration / %	38.3 (35.88, 40.68)
Bilayer roughness / Å	4.93 (4.48, 4.94)
Tail-group thickness / Å	27.48 (27.24, 27.8)
h-Tail-group hydration / %	3.19 (2.27, 4.35)
d-Tail-group hydration / %	0.63 (0.02, 1.75)

Supplementary Table 6: Fitting parameters for the DMPC bilayer in presence of **(*S*)-1** measured on the reflectometer INTER at 37°C. The 95% confidence intervals obtained from the error analysis is shown in brackets.

Fitting parameter	Fitted value
Substrate roughness / Å	5.28 (4.05, 6.48)
Silicon oxide thickness / Å	12.98 (12.32, 13.66)
Silicon oxide roughness / Å	7.90 (7.69, 8.00)
Silicon oxide hydration / %	15.42 (12.3, 18.27)
Head-group thickness / Å	8.45 (7.89, 9.03)
d-Head-group hydration / %	42.97 (39.67, 46.12)
Bilayer roughness / Å	4.25 (3.89, 4.54)
Tail-group thickness / Å	27.43 (27.12, 27.89)
h-Tail-group hydration / %	2.43 (1.31, 3.56)
d-Tail-group hydration / %	0.43 (0.14, 1.01)
Peptide fraction in head-group	0.07 (0.03, 0.15)
Peptide fraction in tail-group	0.03 (0.03, 0.04)

Supplementary Table 7: Fitting parameters for the DMPC bilayer in presence of **(*R*)-1** measured on the reflectometer INTER at 37°C. The 95% confidence intervals obtained from the error analysis is shown in brackets.

Fitting parameter	Fitted value
Substrate roughness / Å	6.26 (4.96, 7.48)
Silicon oxide thickness / Å	11.29 (10.25, 12.17)
Silicon oxide roughness / Å	7.75 (7.24, 7.99)
Silicon oxide hydration / %	13.64 (8.49, 18.02)
Head-group thickness / Å	9.64 (9.09, 10.15)
d-Head-group hydration / %	45.49 (39.74, 51.93)
Bilayer roughness / Å	4.65 (4.24, 4.99)
Tail-group thickness / Å	27.49 (26.87, 28.25)
h-Tail-group hydration / %	0.04 (0.00, 0.15)
d-Tail-group hydration / %	0.58 (0.23, 0.85)
Peptide fraction in head-group	0.05 (0.03, 0.08)
Peptide fraction in tail-group	0.05 (0.03, 0.29)

Supplementary Table 8: Fitting parameters for the DMPC bilayer in presence of ***rac*-1** 9% w/w measured on the reflectometer INTER at 37°C. The 95% confidence intervals obtained from the error analysis is shown in brackets.

Fitting parameter	Fitted value
Substrate roughness / Å	4.54 (3.39, 5.59)
Silicon oxide thickness / Å	13.42 (12.77, 14.05)
Silicon oxide roughness / Å	7.73 (7.19, 7.99)
Silicon oxide hydration / %	14.01 (10.99, 16.72)
Head-group thickness / Å	9.37 (8.72, 10.10)
d-Head-group hydration / %	44.12 (40.67, 47.35)
Bilayer roughness / Å	3.40 (2.63, 4.17)
Tail-group thickness / Å	28.63 (27.71, 29.42)
h-Tail-group hydration / %	6.19 (4.30, 7.72)
d-Tail-group hydration / %	3.78 (2.00, 5.18)
Peptide fraction in head-group	0.09 (0.02, 0.19)
Peptide fraction in tail-group	0.04 (0.02, 0.06)

Supplementary Table 9: Fitting parameters for the DMPC bilayer in presence of **rac-1** 17% w/w measured on the reflectometer INTER at 37°C. The 95% confidence intervals obtained from the error analysis is shown in brackets.

Fitting parameter	Fitted value
Substrate roughness / Å	4.54 (3.39, 5.59)
Silicon oxide thickness / Å	13.93 (13.33, 15.53)
Silicon oxide roughness / Å	7.81 (7.25, 7.99)
Silicon oxide hydration / %	19.95 (17.18, 23.09)
Head-group thickness / Å	10.23 (9.81, 10.78)
d-Head-group hydration / %	43.79 (39.96, 47.42)
Bilayer roughness / Å	4.83 (4.30, 5.28)
Tail-group thickness / Å	25.92 (24.95, 26.96)
h-Tail-group hydration / %	1.54 (0.75, 2.39)
d-Tail-group hydration / %	0.04 (0.00, 0.11)
Peptide fraction in head-group	0.06 (0.02, 0.09)
Peptide fraction in tail-group	0.11 (0.02, 0.20)

S.2.1 Supplementary Figures

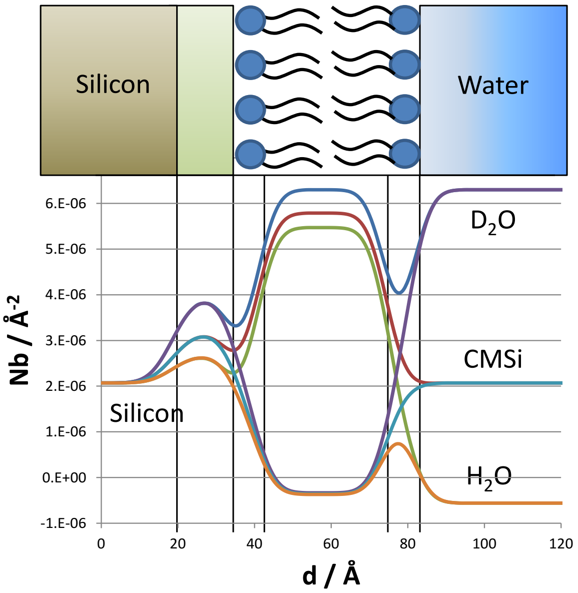

Supplementary Figure 4: Schematic diagram showing the contrasts used in the NR experiments

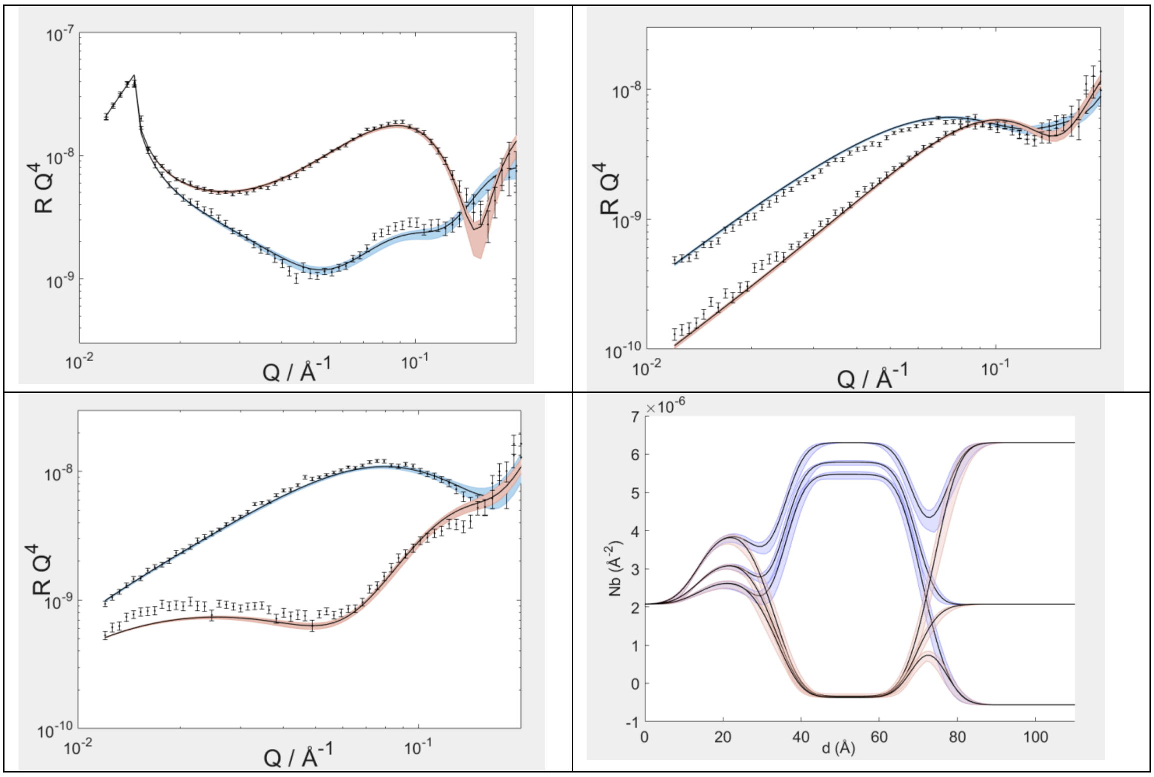

Supplementary Figure 5: Neutron reflectivity profiles and best fit lines for DMPC bilayer measured in (a) D2O, (b) Silicon Matched water and (c) H2O. The corresponding scattering length density profiles are shown in (d). In all plots the h-lipid is represented by the red fit and the d-lipid by the blue line.

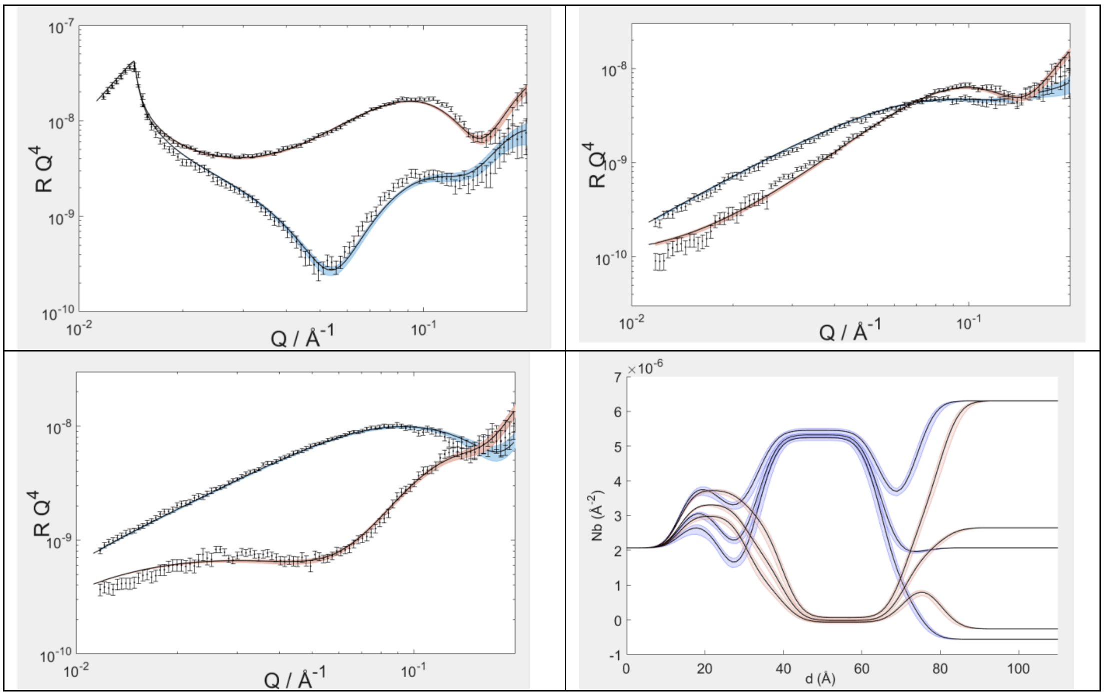

Supplementary Figure 6: Neutron reflectivity profiles and best fit lines for DMPC bilayer in presence of peptide **2** measured in (a) D_2O, (b) Silicon Matched water and (c) H_2O. The corresponding scattering length density profiles are shown in (d). In all plots the h-lipid is represented by the red fit and the d-lipid by the blue line. All fitting parameters are shown in Supplementary Table 2.

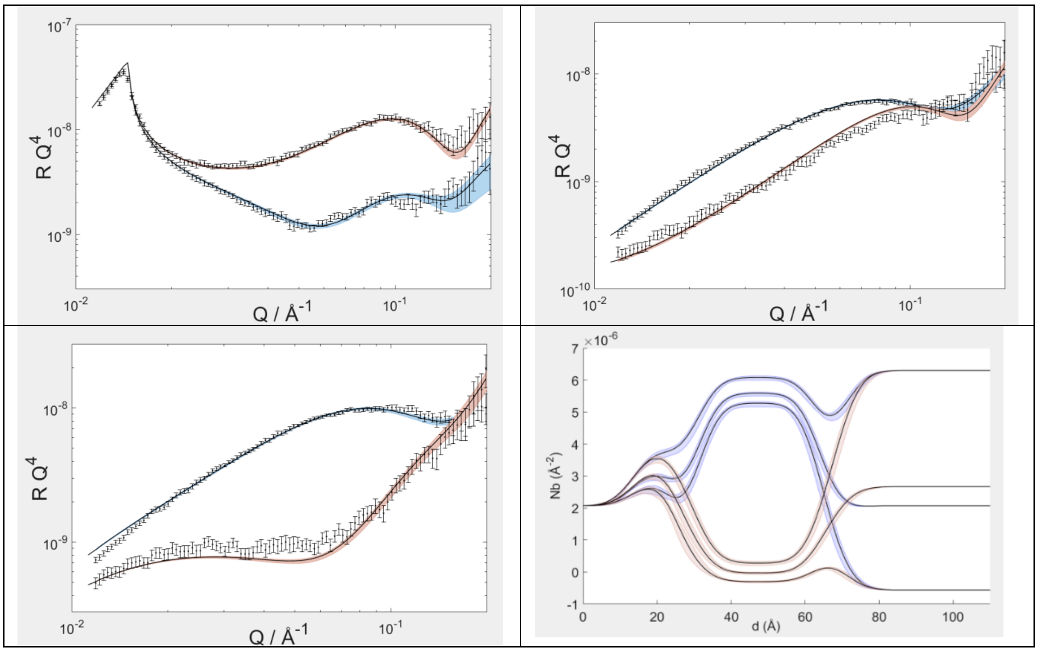

Supplementary Figure 7: Neutron reflectivity profiles and best fit lines for DMPC bilayer in presence of peptide **3** measured in (a) D_2O, (b) Silicon Matched water and (c) H_2O. The corresponding scattering length density profiles are shown in (d). In all plots the h-lipid is represented by the red fit and the d-lipid by the blue line. All fitting parameters are shown in Supplementary Table 3.

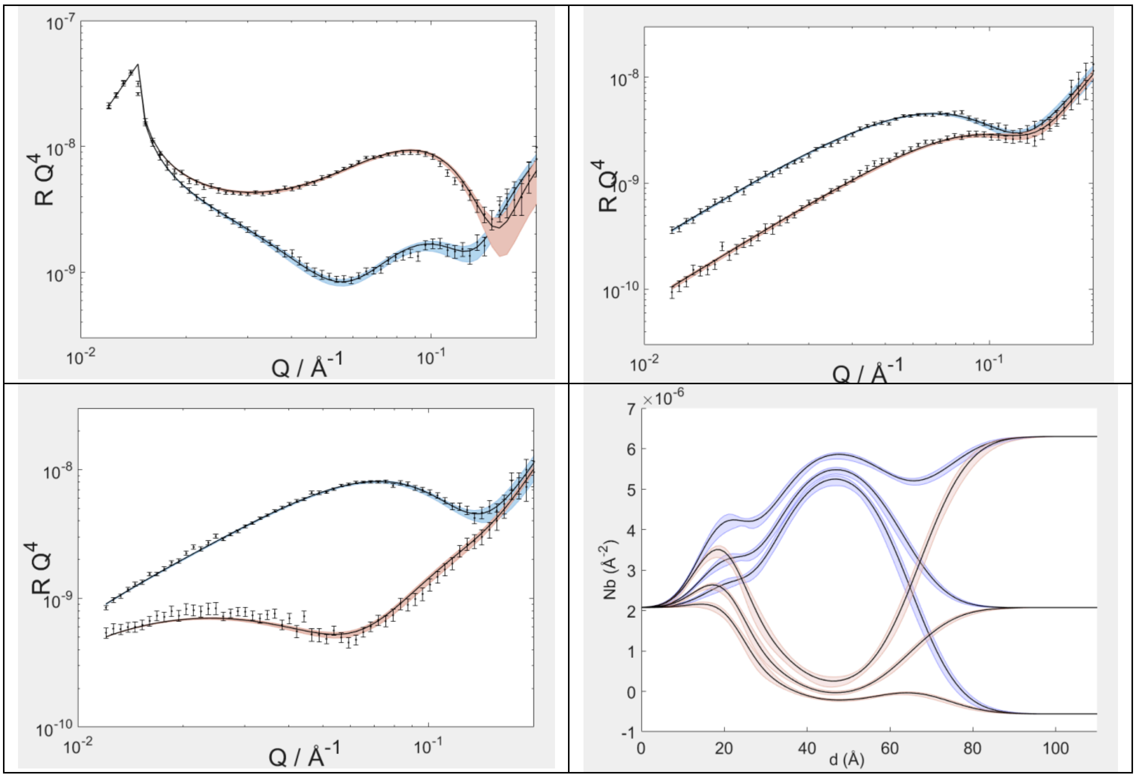

Supplementary Figure 8: Neutron reflectivity profiles and best fit lines for DMPC bilayer in presence of peptide **(*S*)-4** measured in (a) D_2O, (b) Silicon Matched water and (c) H_2O. The corresponding scattering length density profiles are shown in (d). In all plots the h-lipid is represented by the red fit and the d-lipid by the blue line. All fitting parameters are shown in Supplementary Table 4.

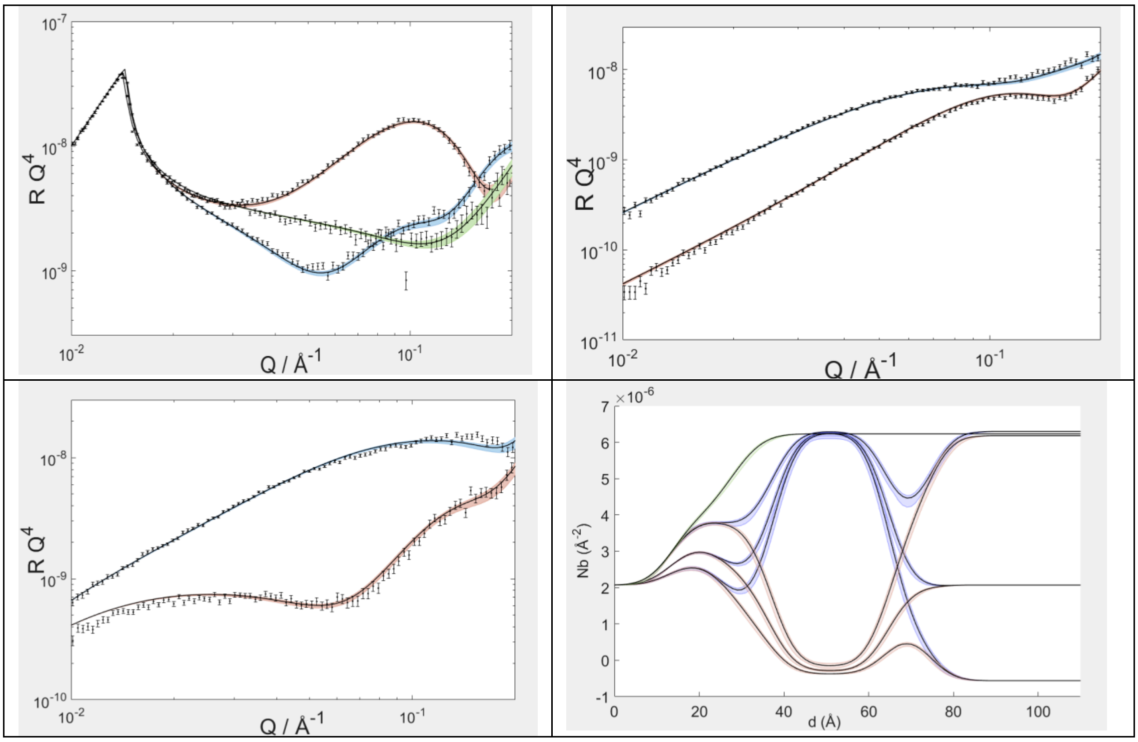

Supplementary Figure 9: Neutron reflectivity profiles and best fit lines for DMPC bilayer measured in (a) D2O, (b) Silicon Matched water and (c) H2O. The corresponding scattering length density profiles are shown in (d). In all plots the h-lipid is represented by the red fit and the d-lipid by the blue line. Bare Silicon/D2O is shown in green. All fitting parameters are shown in Supplementary Table 5.

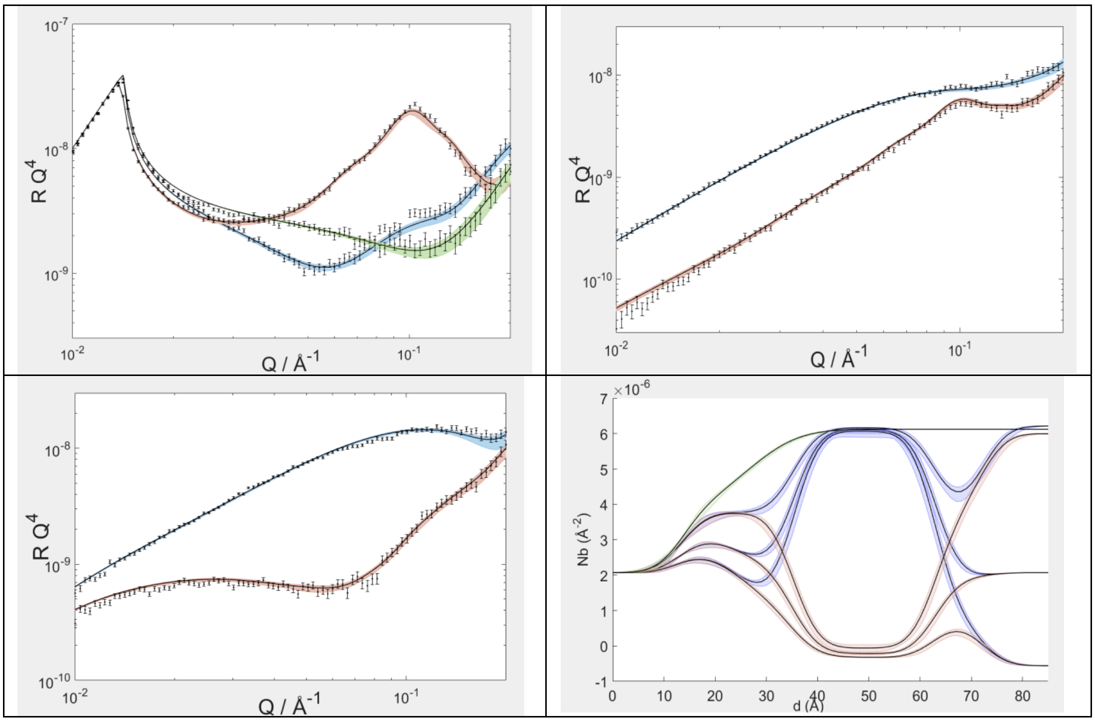

Supplementary Figure 10: Neutron reflectivity profiles and best fit lines for DMPC bilayer in presence of ***(S)*-1** measured in (a) D_2O, (b) Silicon Matched water and (c) H_2O. The corresponding scattering length density profiles are shown in (d). In all plots the h-lipid is represented by the red fit and the d-lipid by the blue line. Bare Silicon/D_2O is shown in green. All fitting parameters are shown in Supplementary Table 6.

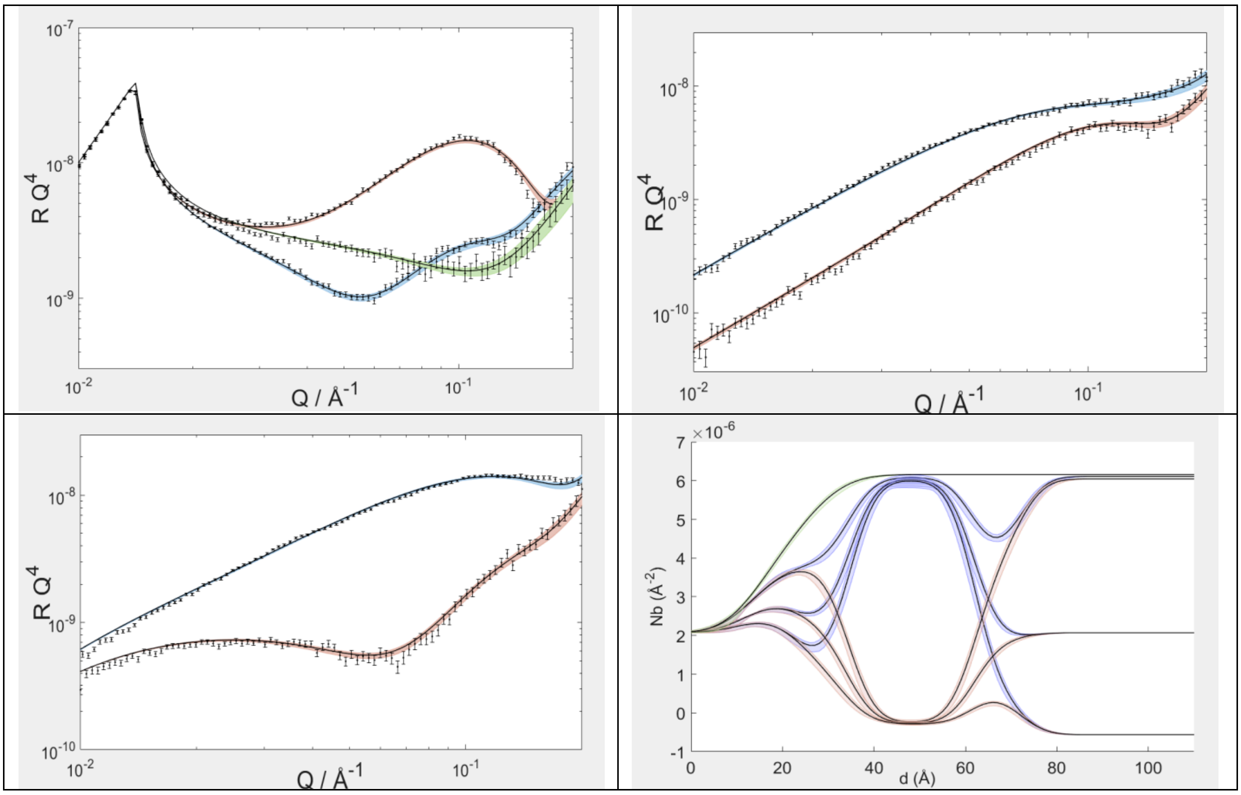

Supplementary Figure 11: Neutron reflectivity profiles and best fit lines for DMPC bilayer in presence of ***(R)*-1** measured in (a) D_2O, (b) Silicon Matched water and (c) H_2O. The corresponding scattering length density profiles are shown in (d). In all plots the h-lipid is represented by the red fit and the d-lipid by the blue line. Bare Silicon/D_2O is shown in green. All fitting parameters are shown in Supplementary Table 7.

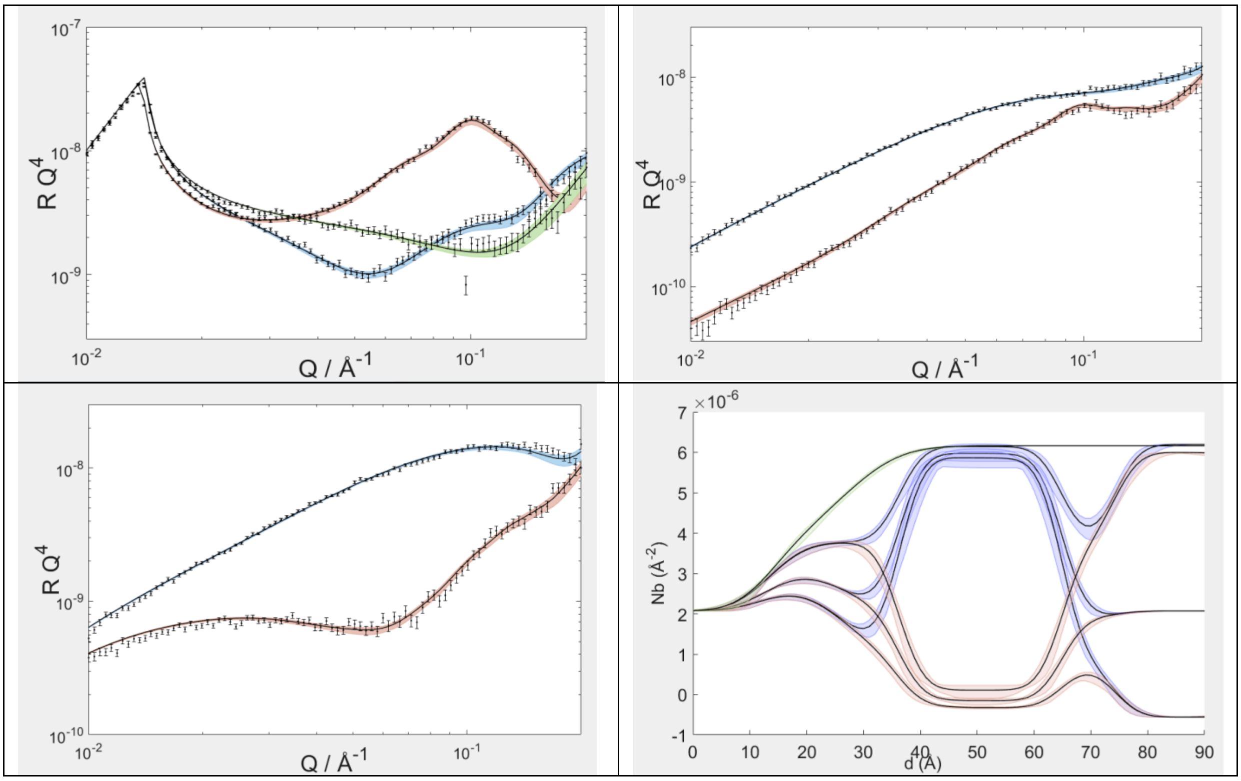

Supplementary Figure 12: Neutron reflectivity profiles and best fit lines for DMPC bilayer in presence of ***rac*-1** 9% w/w measured in (a) D_2O, (b) Silicon Matched water and (c) H_2O. The corresponding scattering length density profiles are shown in (d). In all plots the h-lipid is represented by the red fit and the d-lipid by the blue line. Bare Silicon/D_2O is shown in green. All fitting parameters are shown in Supplementary Table 8.

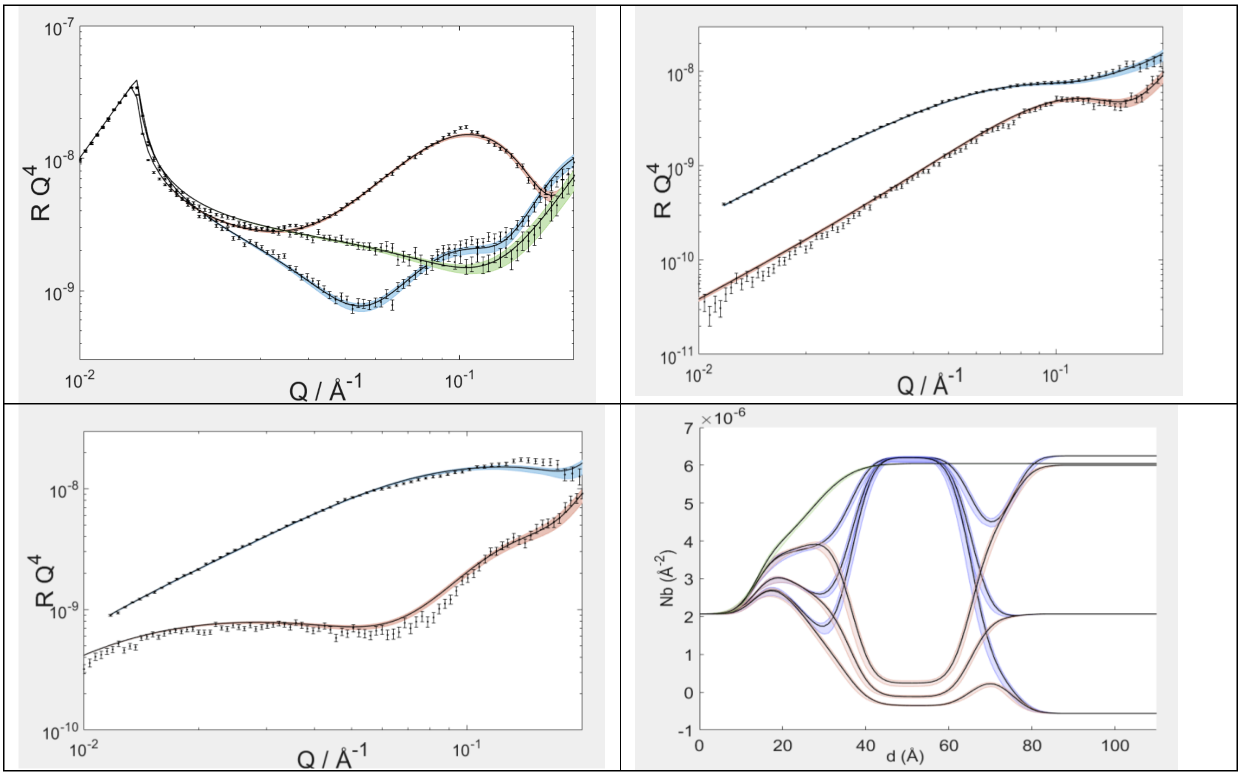

Supplementary Figure 13: Neutron reflectivity profiles and best fit lines for DMPC bilayer in presence of ***rac*-1** 17% w/w measured in (a) D_2O, (b) Silicon Matched water and (c) H_2O. The corresponding scattering length density profiles are shown in (d). In all plots the h-lipid is represented by the red fit and the d-lipid by the blue line. Bare Silicon/D_2O is shown in green. All fitting parameters are shown in Supplementary Table 9.

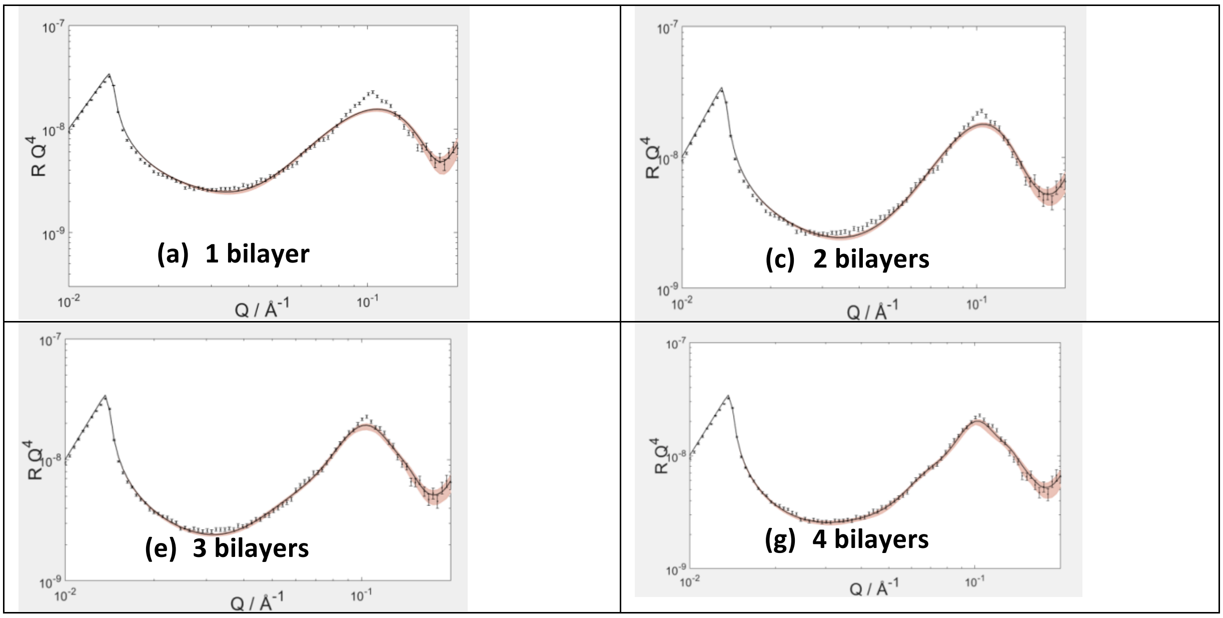

Supplementary Figure 14: Reflectivity profile for h-DMPC bilayer with **(*S*)-1** 7% w/w at the Silicon/D2O interface and best line fit with a single bilayer is shown in (a). The peak at Q ~0.1 Å^{-1} is indication of multilayer patches and cannot be fitted unless more layers are added to the model. A minimum of 4 bilayers are required to adequately fit the peak. Whereas the coverage of the primary bilayer is 97.6%, the additional bilayers form small patches with coverage of 9.0, 4.2 and 3.7% for the 2nd, 3rd and 4th bilayer respectively.

S.4 Molecular dynamics simulations

S.4.1 Supplementary Table: Average angles

Supplementary Table 10: Average angles over the final 100/50 ns of the simulations between the membrane normal and the short (270 nm) and long (210 nm) axes of the benzyl groups in the monomer and dimer **(*S*)-1 molecular dynamics** membrane simulations. The quoted range is the standard deviation. Mid refers to the mid-chain benzyl group and terminal to the terminal benzyl. The table shows both simulation and experimental data.

	**Terminal long 210 nm**	**Mid long 210 nm**	**Terminal short 270 nm**	**Mid short 270 nm**
Monomer simulation	59 ± 12°	59 ± 11°	52 ± 12°	58 ± 11°
Dimer simulation	48 ± 13°<br>49 ± 12°	63 ± 11°<br>58 ± 9°	53 ± 7°<br>53 ± 7°	47 ± 12°<br>54 ± 11°<br>(>60° for final 50 ns)
Experiment **(*S*)-2**	+	N/A	+	N/A
Experiment mid + terminal **(*S*)-1**	>54.7°		>54.7°	

S.4.2 Supplementary Figures

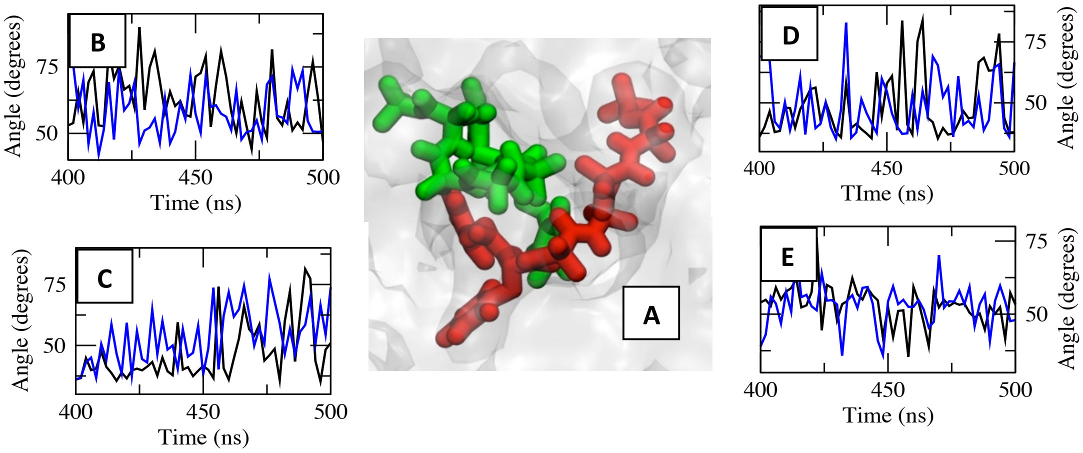

Supplementary Figure 15: (A) Final frame, top-view snapshot of the ***S*-(1)** dimer (red and green monomers) embedded in the DOPC bilayer (translucent white surface) with water molecules omitted for clarity. (B, C) Time series for the long (B) and short (C) axis angles for the mid-chain phenyl rings relative to the membrane normal. (D, E) Time series for the long (D) and short (E) axis angles for the terminal phenyl rings relative to the membrane normal. Black lines represent data for one CbzPheAib$_4$O^{t}Bu molecule and blue lines represent data for the second CbzPheAib$_4$O^{t}Bu molecule.
